# Social contact patterns relevant for infectious disease transmission in Cambodia

**DOI:** 10.1038/s41598-023-31485-z

**Published:** 2023-04-04

**Authors:** William T. M. Leung, Aronrag Meeyai, Hannah R. Holt, Borin Khieu, Ty Chhay, Sokeyra Seng, Samkol Pok, Phiny Chiv, Tom Drake, James W. Rudge

**Affiliations:** 1 Communicable Diseases Policy Research Group, Department of Global Health and Development, London School of Hygiene and Tropical Medicine, Keppel St, London, WC1E 7HT UK; 2 Department of Epidemiology, Faculty of Mahidol Public Health, Mahidol University, Bangkok, 10400 Thailand; 3 Centre for Tropical Medicine and Global Health, Nuffield Department of Medicine, University of Oxford, Oxford, OX3 7LG UK; 4 Livestock Development for Community Livelihood Organization, St 181, Phnom Penh, Cambodia; 5 National Institute of Science, Technology and Innovation, Ministry of Industry, Science, Technology and Innovation, National Road 2, Phnom Penh, Cambodia

**Keywords:** Public health, Epidemiology

## Abstract

Social mixing patterns are key determinants of infectious disease transmission. Mathematical models parameterised with empirical data from contact pattern surveys have played an important role in understanding epidemic dynamics and informing control strategies, including for SARS-CoV-2. However, there is a paucity of data on social mixing patterns in many settings. We conducted a community-based survey in Cambodia in 2012 to characterise mixing patterns and generate setting-specific contact matrices according to age and urban/rural populations. Data were collected using a diary-based approach from 2016 participants, selected by stratified random sampling. Contact patterns were highly age-assortative, with clear intergenerational mixing between household members. Both home and school were high-intensity contact settings, with 27.7% of contacts occurring at home with non-household members. Social mixing patterns differed between rural and urban residents; rural participants tended to have more intergenerational mixing, and a higher number of contacts outside of home, work or school. Participants had low spatial mobility, with 88% of contacts occurring within 1 km of the participants’ homes. These data broaden the evidence-base on social mixing patterns in low and middle-income countries and Southeast Asia, and highlight within-country heterogeneities which may be important to consider when modelling the dynamics of pathogens transmitted via close contact.

## Introduction

Infectious diseases spread via close contact exert a huge burden on health systems, local and global economies and societies. Mathematical models of infectious diseases have been used extensively to elucidate transmission dynamics, evaluate control strategies, and inform policy^1^. Central to these models are transmission parameters, including the effective contact rate between individuals or groups describing population mixing. Models of directly-transmitted pathogens commonly assume that transmission rates are proportional to the rate of contact between individuals, and often explicitly incorporate age-specific data on social mixing patterns^2,3^. Models allowing for heterogeneous contact rates between age groups have described age-specific infection rates for pathogens including influenza, pertussis and mumps^3–5^. In addition to the number of contacts, the intensity, duration, and location of contacts can also influence person-to-person transmission dynamics^6^.

Consideration of social mixing patterns is crucial for models aiming to simulate the impact of non-pharmaceutical interventions (NPIs) such as school and workplace closures, elderly shielding, and other social distancing measures^7^. This has been brought into sharp focus recently, as governments have implemented unprecedented measures as part of the SARS-CoV-2 pandemic response, often with guidance from models incorporating empirical data on social mixing patterns^8–12^.

Although patterns of mixing are critical determinants of infectious disease transmission, there is a lack of data on social mixing patterns in many countries^2,7^. The landmark POLYMOD project by Mossong et al.^13^ surveyed, for the first time, contemporary mixing patterns in eight European countries. Using a contact diary approach, the study collected information on approximately 100,000 contacts of 7290 participants, including their age, duration, nature (physical/non-physical), and setting^13^. Data from this study continues to be used in many transmission modelling studies. Since then, empirical contact data has been collected in a number of other geographical regions^14–18^.

However, a 2019 systematic review of social contact surveys found that 80% of studies were conducted in High Income Countries and, despite being the most populous continent, covered only five Asian countries^2^. Furthermore, only three studies included in the review examined differences between (peri)-urban and rural populations^16,19,20^. Demographic and social structures, socio-cultural norms, population mobility and occupations vary substantially between (and even within) countries, and there is a need for surveys describing heterogenous contact patterns in diverse regions^2,21^. To address geographical gaps in empirical data, ‘synthetic’ contact matrices have been projected for other countries using available demographic data until such data are available^7^.

South-east Asia is recognised as hotspot for emergence and spread of infectious diseases, including those with pandemic potential. This is driven by factors such as increasing population sizes and mobility, coupled with environmental and land-use change including urbanisation, deforestation and intensification of livestock systems^22^. To contribute to the evidence-base on social mixing patterns in different contexts, here we present an in-depth analysis of data from a community-based contact survey in Cambodia conducted in 2012. The study aimed to characterise mixing patterns and spatial mobility, generate setting-specific contact matrices between age-groups, and assess how these patterns may vary between urban and rural populations.

## Methods

### Study design and population

**Figure 1:**
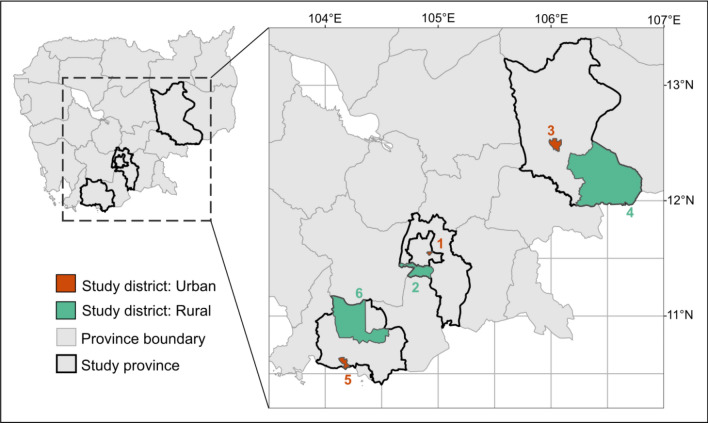
Map of the six study districts within Cambodia. Orange = urban, green = rural. (1) Chamka Morn, Phnom Penh municipality; (2) Kandal Stung, Kandal Province; (3) Krong Kracheh, Kratie Province; (4) Snuol, Kratie Province; (5) Krong Kampot, Kampot Province; (6) Chhuk, Kampot Province. The borders of included provinces are marked with heavy black lines. Within each selected district, three sub-administrative divisions (‘sangkats’—urban; ‘communes’—rural) were randomly selected.

Data were collected through a cross-sectional survey in May 2012, and employed a multi-stage, quota sampling strategy. In the first stage, three main study areas were purposefully selected to enhance geographic representativeness, whilst keeping the geographic scope logistically feasible. These study areas were: (i) Phnom Penh municipality (the capital) and surrounding Kandal province, located in south-central Cambodia, which informally comprise the Phnom Penh greater metropolitan area; (ii) Kampot province (south-west Cambodia); and (iii) Kratie province (east Cambodia) (Fig. 1). Within each province, one rural and one urban district (as designated by national census data) were randomly selected. Three communes were then randomly selected from each study district.

The total target sample size was 2016 people, aiming for equal numbers of rural and urban residents. The sampling strategy was further stratified to target equal ratios of study participants by study district, gender (male and female) and age-group (using the age bands: 0–5, 6–12, 13–19, 20–29, 30–39, 40–59, 60 +), giving a sampling quota of 72 participants within each demographic (age-gender-urban/rural) stratum. This sampling strategy, along with the data collection tools described below, were designed based on those developed by the consortium of another project (Social Mixing for Influenza-Like Illness in Asia; PI: John Edmunds) involving contact surveys in other Asian countries.

Study participants were recruited through door-to-door household visits. Households were selected by picking a random direction (determined by spinning a pen) from the approximate centre of each commune and visiting each successive house in that direction. When someone was at home and consented to participate, a single household member was recruited, providing the consenting person was within a demographic stratum for which the quota had not been reached.

### Data collection

Upon recruitment, a background questionnaire was administered to collect data on demographic and occupational variables, household structure and usual travel frequencies outside of their administrative divisions. Social contact data were collected following similar methods used by Mossong et al*.*^13^. Participants were prospectively allocated a 24-h period in which they were instructed to remember all contacts they made beginning at 3 a.m. on the morning of the assigned day, where a ‘contact’ was defined as either “a two-way conversation in the physical presence of another person” or “physical skin-to-skin contact”. Participants were provided with a contact diary and encouraged to record every contact they made throughout their allocated day. An interviewer also led them through its completion the following day using a semi-structured series of prompts to aid recall.

At both visits (i.e. at recruitment and during the follow-up visit after the diary day), it was emphasised to study participants that they should try to capture all contacts within the contact diary. However, it was also acknowledged that some participants may have a large number of contacts, and/or “casual” contacts, which might result in underreporting of contacts in the contact diary. Therefore, after reviewing the diary with the participant, the interviewer also asked the participant whether they thought any contacts had been omitted in the diary. If so, participants were then asked to estimate the number of additional physical or non-physical contacts (using the same definition described above) in each of four age bands representing children (0–4 and 5–15 years), adults (16–64 years), and seniors (aged 65 +). These additional contacts are henceforth referred to as ‘supplementary contacts’, as opposed to ‘diary-reported contacts’.

### Ethical approval

This study was approved by the London School of Hygiene and Tropical Medicine (LSHTM) Institutional Review Board (Ref: 6129), and by the Cambodia Ministry of Health National Ethics Committee for Health Research (Ref: 188). All methods were performed in accordance with the relevant guidelines and regulations. Written informed consent was obtained from all study participants, or from a parent/guardian if the participant was under 18 years of age.

### Data analysis

#### Number and duration of contacts

All analyses were conducted using R^23^. Sampling weights generated by raking were applied to adjust the distribution of selected variables towards population levels (Table S1; supplementary)^24^. The following variables were used for weighting: urban/rural, age group, weekday/weekend, and employment status based on three levels: non-working age, employed, and unemployed, as defined by the Cambodian Socioeconomic Survey 2013 (CSES; National Institute of Statistics, 2013).

We estimated the effects of covariates of interest on the total number of contacts (a participant’s “degree”) and the total contact hours using multivariable negative binomial regression. Total contact hours were estimated by summing the contact durations across all contacts reported in the diary. Since duration of contact was reported in one of five ranges (< 5 min, 5–14 min, 15–59 min, 1–4 h, or > 4 h), the mid-point of each range was used, right truncated at 4 h^18,25^. Analysis of total contact hours was restricted to diary-reported contacts, as contact duration was not collected for supplementary contacts.

#### Age-specific mixing

Age-structured mixing matrices were generated using five-year age bands from 0 to 64 years, and a single age band for participants aged 65 and over. Different matrices (showing the mean number of contacts an individual in age group $i$ made with an individual in age group $j$) were generated for urban and rural participants in each of five settings: (1) at home with household members, (2) at home with non-household members, (3) at work, (4) at school or college, (5) on transport, during leisure time, or in other undefined settings. Household members were defined as “those which share a living space with the participant and use the same doorway exiting to a public space”. Only people for whom the address is their main residence were included. To observe the age-specific mixing patterns of high-intensity contacts, another set of matrices were generated for contacts that were either physical in nature, or ≥ 15 min in duration. Supplementary contacts were not included in the mixing matrices because the settings, and precise ages, were not collected for these contacts.

All matrices were weighted using the variables described previously and were adjusted to account for the reciprocal nature of contacts^26^. That is, the total number of contacts that age group ($i$) made with age group ($j$), should be equal to the total number of contacts age group ($j$) made with age group ($i$). Mean contact rates were adjusted using Formula (1)

1 $${c\mathrm{^{\prime}}}_{ij}=\frac{1}{2{N}_{i}}\left({c}_{ij}{N}_{i}+{c}_{ji}{N}_{j}\right), \,\mathrm{ where} \,{c}_{ij}{N}_{i}={c}_{ji}{N}_{j}$$

where ${c}_{ij}$ or ${c}_{ji}$ is the mean contact rates between age groups, ${N}_{i}$ or ${N}_{j}$ is the population size of age group $i$ and $j$, respectively. Population sizes were extracted from the Cambodian Socioeconomic Survey 2013 (CSES; National Institute of Statistics, 2013).

#### Comparison of reporting methods

To assess for potential bias in the reporting of contacts, comparisons were made between the age distributions, and intensity (physical vs non-physical) of contacts reported in the contact diary vs those reported as supplementary contacts. Comparisons were made using Pearson’s chi squared tests with^27^.

## Results

### Description of the sampled participants

Data were obtained from 2016 study participants in May 2012, with equal target ratios achieved for each gender, age-group, and urban/rural locations (Table S1). The median age of surveyed participants was 24 years (range: 4 days to 93 years), and 85.2% of diaries were completed on a weekday (Mon–Fri). Among participants aged between 15 and 64 years, employment rates were lower than those reported in the Cambodian Socioeconomic Survey of the same year for urban areas (64.0% vs. 86.1%) but comparable for rural areas (77.8% vs. 79.6%) (National Institute of Statistics, 2013).

### Number and duration of contacts

A combined total of 59,597 (diary and supplementary) contacts were reported across all study participants, with half of these being recorded in the contact diary (29,358; 49%). In total, this equated to a weighted median of 22 (Interquartile range [IQR]: 13–40) contacts recorded per person day: 13 (IQR: 8–20) diary-based contacts, and 7 (IQR: 0–21) supplementary contacts (Table S2). Participant age, occupation, urban/rural residence, and day of the week the diary was completed, were all significantly associated with the number of contacts made (ANOVA p < 0.001, Table 1). Meanwhile, age, occupation, urban/rural designation, household size, and the number of rooms were significantly associated with total contact hours. The number and duration of contacts did not differ significantly between male and female participants.

**Table 1:** Relative difference in degree (number of contacts), and total contact hours according to multivariable negative binomial regression; Significance: ***P < 0.001; **P < 0.01; *P < 0.05.

		N (%)	Relative degree (95% CI)	ANOVA	Relative hours contact (95% CI)	ANOVA
	(Intercept)		18.77 (15.96–22.11) ***		19.01 (16.54–21.88) ***	
Urban/rural	Urban	1008 (50)	REF	***	REF	***
	Rural	1008 (50)	1.23 (1.17–1.3) ***		1.12 (1.07–1.17) ***	
Day of the week	Weekday	1717 (85.2)	REF	***	REF	0.059
	Weekend	299 (14.8)	0.81 (0.74–0.89) ***		1 (0.92–1.08)	
Sex	Male	1006 (49.9)	REF	0.91	REF	0.48
	Female	1007 (50)	1 (0.93–1.06)		0.98 (0.92–1.03)	
Household size	*Per unit increase*	–	1 (0.98–1.02)	0.27	1.1 (1.08–1.12) ***	***
Number of rooms	1	1150 (57)	REF	0.29	REF	***
	2	472 (23.4)	0.98 (0.9–1.06)		0.91 (0.85–0.98) **	
	3	212 (10.5)	1.02 (0.91–1.14)		0.85 (0.77–0.94) ***	
	4	111 (5.5)	0.93 (0.8–1.08)		0.83 (0.73–0.95) **	
	≥ 5	69 (3.4)	1.05 (0.88–1.27)		0.81 (0.7–0.96) *	
Age group	0–4	231 (11.5)	REF	***	REF	***
	5–14	440 (21.9)	1.04 (0.87–1.25)		1.21 (1.04–1.42) *	
	15–24	355 (17.6)	1.35 (1.1–1.66) **		1.14 (0.95–1.37)	
	25–34	278 (13.8)	1.35 (1.07–1.71) *		1.02 (0.83–1.26)	
	35–44	224 (11.1)	1.32 (1.04–1.68) *		0.86 (0.7–1.07)	
	45–54	137 (6.8)	1.17 (0.91–1.5)		0.88 (0.71–1.11)	
	55–64	174 (8.6)	1.11 (0.87–1.42)		0.75 (0.61–0.94) **	
	≥ 65	177 (8.8)	0.77 (0.6–0.99) *		0.69 (0.55–0.86) ***	
Occupation	Child at home	286 (14.2)	REF	***	REF	***
	Pre-schooler	75 (3.7)	1.51 (1.22–1.87) ***		1.27 (1.05–1.53) *	
	School/college student	466 (23.1)	1.47 (1.24–1.75) ***		1.06 (0.91–1.23)	
	Professional/office worker	84 (4.2)	1.64 (1.27–2.13) ***		1.07 (0.85–1.34)	
	Shop worker/trader	165 (8.2)	1.98 (1.57–2.49) ***		0.87 (0.7–1.06)	
	Manual labourer, non-agri	131 (6.5)	1.29 (1.02–1.64) *		0.84 (0.68–1.04)	
	Agriculture and fishing	459 (22.8)	1.29 (1.04–1.59) *		1.05 (0.87–1.27)	
	Housewife	134 (6.6)	1.15 (0.9–1.46)		0.97 (0.78–1.2)	
	Retired	54 (2.7)	1.34 (1–1.8)		0.95 (0.73–1.24)	
	Unemployed	95 (4.7)	1.4 (1.09–1.8) **		0.94 (0.75–1.17)	
	Others	64 (3.2)	2 (1.54–2.61) ***		1.08 (0.85–1.37)	

Fewer contacts were reported by people completing their contact diaries on weekends; however, there was no significant difference in cumulative contact duration suggesting that on these days, people have fewer, but longer duration contacts.

Apart from housewives or retirees, all occupational groups had significantly greater relative degree compared to the reference group of children at home. Shop workers/traders had approximately twice the number of contacts than children at home. Despite this wide variability, there was little difference in the total contact hours made by different occupational groups. Total contact hours were highest in pre-schoolers.

### Age-specific mixing

**Figure 2:**
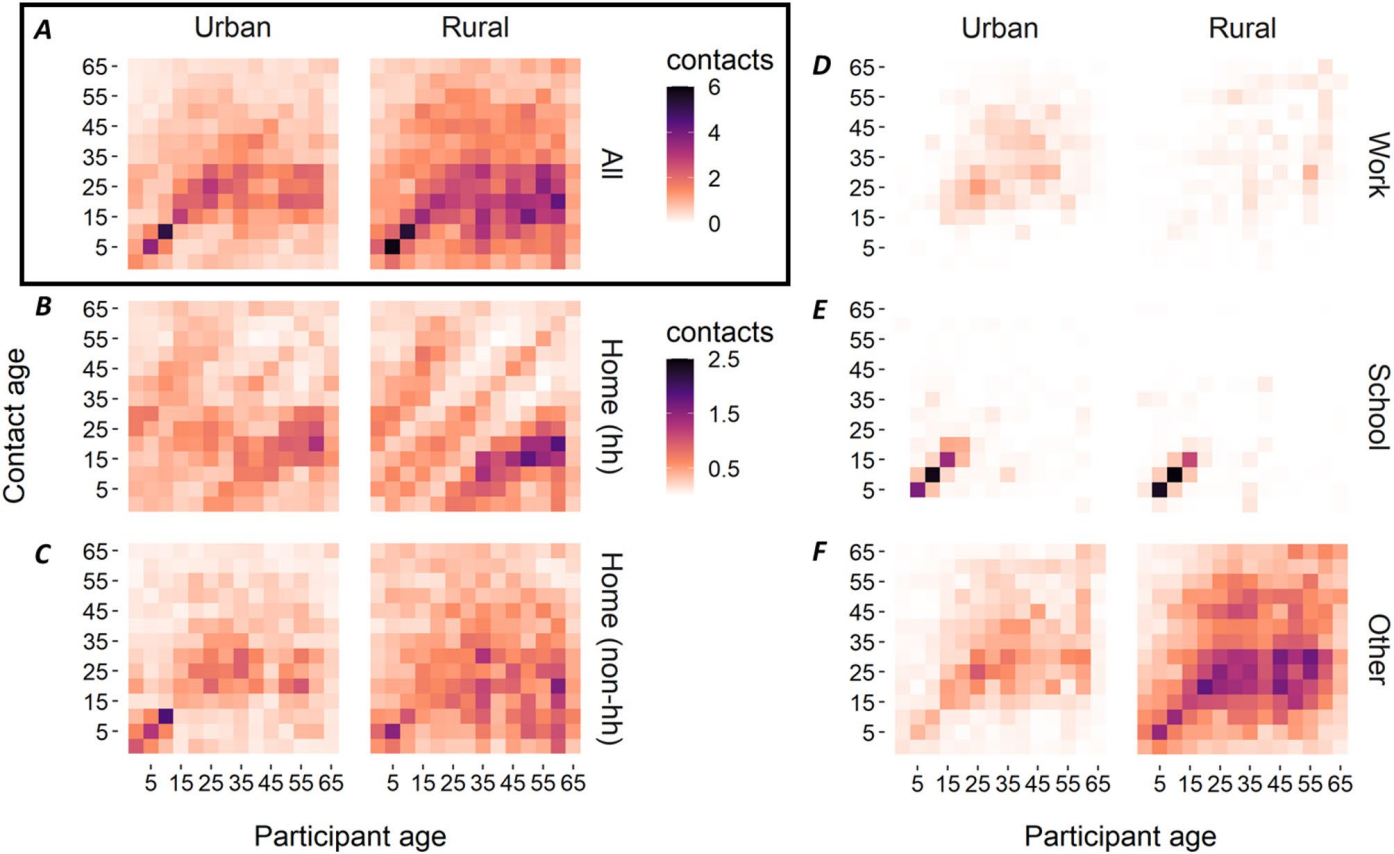
Contact matrices stratified by urban and rural (participant residence locations, (**A–F**), and by social setting (**B–F**). Each cell in a given matrix shows the mean number of contacts made between two age groups. All matrices are weighted by day of the week (weekday or weekend) and employment status (non-working age, employed, or unemployed). Matrices were further adjusted to account for recipricocity of contacts such that the total number of contacts that age group (i) made with age group (j), were equal to the total number of contacts age group (j) made with age group (i) (c_ij_N_i_ = c_ji_N_j_; where N is the population size of an age group). Home (hh) = contacts at home with household members; Home(non-hh) = contacts at home with non-household members. Contact matrices which included only contacts that were physical or over 15 min in duration (which accounted for 81.2% of all diary-reported contacts) exhibited the same patterns of age-specific mixing (Fig. S1A–F).

Age-assortative mixing (i.e., mixing between people of similar ages) was apparent in both urban and rural populations, as shown by the central diagonal lines in the matrices which include contacts across all settings (Fig. 2A, see Table S3 for numeric matrices). This was most pronounced in school-aged children (5–14 years). Older participants (aged between around 35 and 64 years) reported more contacts on average with younger people (aged 5–34), than was reported in the other direction (Fig. 2A). This was particularly pronounced in rural locations (Fig. 2A), and is consistent with the relatively young demographic structure in these locations, as with the country as a whole (Fig. S5).

Most intergenerational mixing occurred at home between household members (Fig. 2B), with the second set of parallel lines representing contacts between generations (e.g. parent–child mixing). Contrastingly, contacts made at home with *non*-household members did not exhibit a clear intergenerational mixing structure (Fig. 2C). At school, the expected strong age-assortative mixing was clear from the age of 5 to 19 years of age in rural locations, and from 5 to 24 years in urban locations (Fig. 2E). Few contacts were reported on transport (0.3%) or during leisure time (0.2%), so these data were combined with the contacts reported in ‘other’ (unspecified) settings for all analyses. In this combined matrix (representing all contacts outside of home, school or work), age-assortative mixing was observed, although there was also substantial mixing across age groups, particularly in rural areas (Fig. 2F).

### Setting of contacts

**Figure 3:**
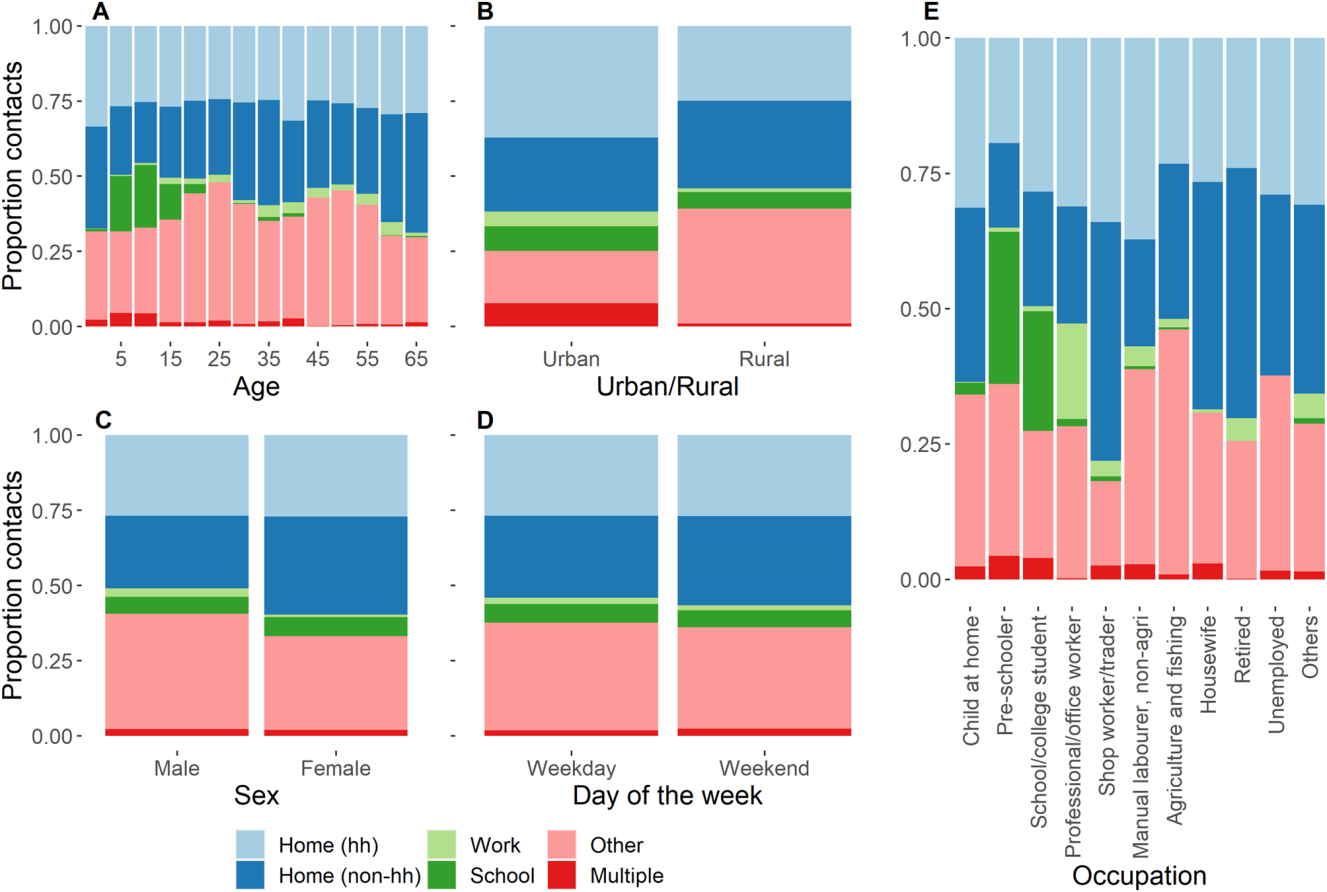
The proportion of contacts reported in each setting according to participant age (**A**), urban or rural residence (**B**), sex (**C**), day of the week (**D**), and occupational group (**E**). ‘Other’ settings refer to contacts made in settings other than home, school, work, or multiple. ‘Multiple’ refers to multiple settings—for example, home and school.

The majority of contacts (56.7%) occurred at home or in ‘other’ settings (that is, other than home, work, or school) (28.5%) (Fig. 3A–E). Of note, over a quarter of all contacts (27.7%) occurred at home with non-household members. Children and young adults (aged 5–19 years) made approximately 20% of their contacts at school/college, while those aged 20–24 made around 10% of their contacts in this setting. The proportion of contacts made at home was highest for children (0–4 years), reached its lowest point at school age (5–19), and had a general trend to increase with age thereafter (Fig. 3A).

The number of contacts made in each setting was significantly associated with participant age (${\chi }^{2}$ = 4318.9, df = 65, p < 0.001), sex (${\chi }^{2}$= 433, df = 5, p < 0.001), urban/rural (${\chi }^{2}$ squared = 2628.4, df = 5, p < 0.001), day of the week (${\chi }^{2}$ = 70.8, df = 5, p < 0.001), and occupational group (${\chi }^{2}$ = 8188.3, df = 50, p < 0.001). Rural participants recorded a greater proportion of their contacts in ‘other’ settings compared with urban participants (37% vs. 16%), and a greater proportion of their home contacts with non-household members (53% vs. 39% of home contacts; Fig. 3B). Though males and females reported no difference in quantity or cumulative duration of contacts (Table 1), the setting where these contacts took place differed, with males reporting more contacts at work and in ‘other’ settings compared to females who made more contacts at home with non-household members (Fig. 3C). Relatively few contacts were reported at work (Fig. 3A–E). Comparing occupational groups, only professional/office workers reported a reasonable proportion of their contacts (18%) in this setting (Fig. 3E). Shop workers and traders reported over 75% of their contacts as ‘Home’ either with household or non-household members.

### Intensity of contacts

**Figure 4:**
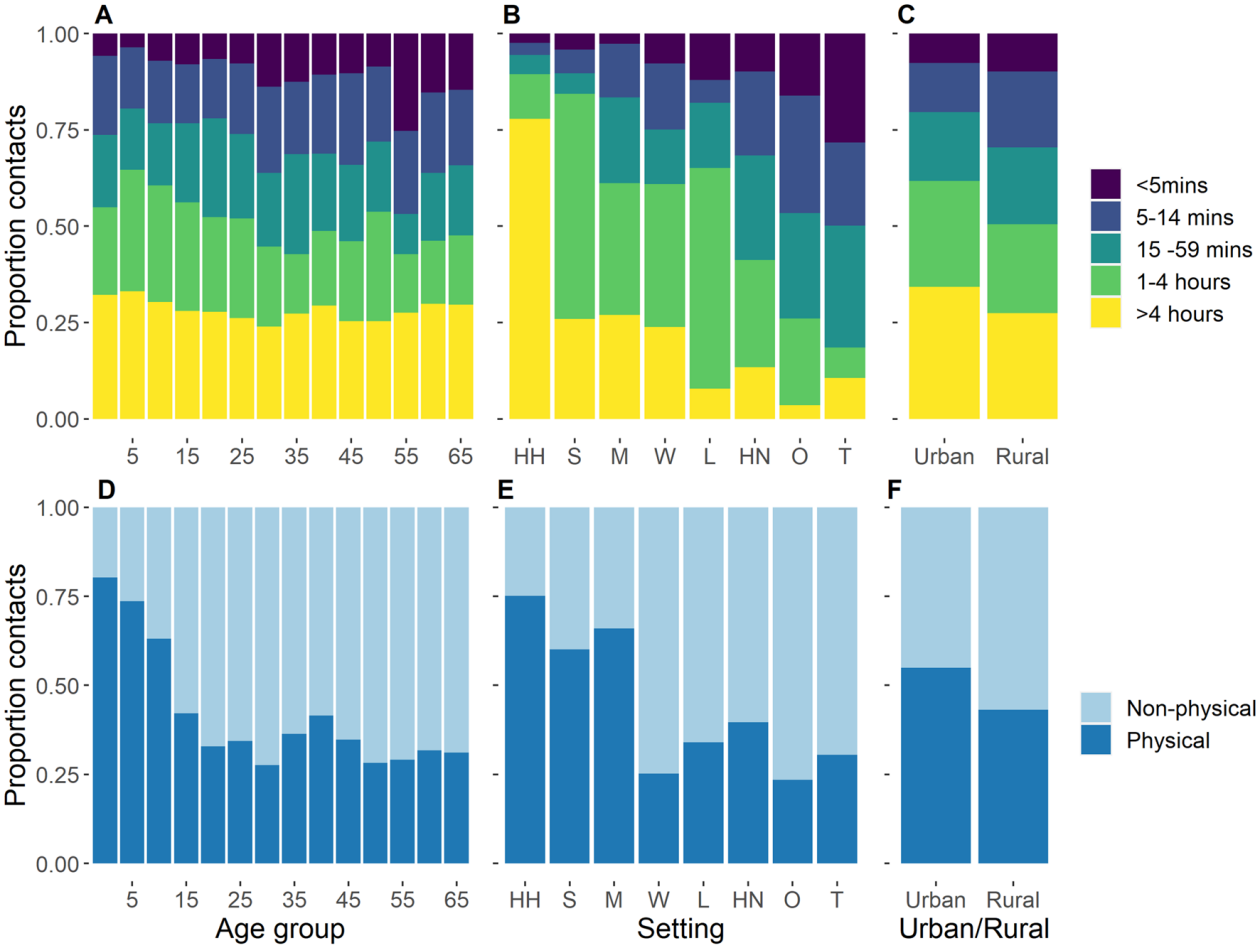
Intensity of contacts, as measured by duration (**A–C**) and proportion physical (**D–F**). Results shown by participant age, social setting (*HH* at home with household members, *S* school, *M* multiple settings, *W* work, *L* leisure, *HN* at home with non-household members, *O* other settings, *T* transport) and urban/rural residence.

Measures of contact intensity were in agreement: as contact duration increased, so too did the likelihood that a contact was physical in nature. Similar patterns were obseved for both of these measures (Fig. S2A; *χ*^2^ test for trend: *χ*^2^ = 5036.9, df = 1, p < 0.001, Fig. 4). More regular contacts were also more likely to be physical; (Fig. S2B; *χ*^2^ = 1529.3, df = 1, p < 0.001). Consistent with regression analysis, there was a general trend for decreasing proportion of physical contacts (Fig. 4A) and duration of contacts (Fig. 4D) with increasing age, up to age group 20 to 24 when this plateaus. The intensity of contacts also varied by setting, with contacts made at home with household members or at school/college more often longer duration, or physical. Contacts recorded in multiple settings were also high intensity owing to the fact that the majority (91%) of these contacts occurred at home (in addition to other settings). While rural participants reported more contacts (Table 1), the contacts of urban participants tended to be of longer duration and were more likely to be physical (Fig. 4C,F).

### Comparison of contact reporting methods

Under half (42%) of participants recorded all of their contacts in the contact diary, with the remainder reporting at least one supplementary contact. The number of reported contacts was right-skewed, especially so for supplementary contacts (Fig. S3). The age distributions of diary-reported contacts vs supplementary contacts differed (${\chi }^{2}$ = 1563.1, df = 3, p < 0.001) with supplementary contacts more likely to be children (0–4 years) and seniors (65 + years) (Fig. S4). When comparing age-contact matrices based on diary contacts and supplementary contacts, using the age bands of the latter, similar patterns were observed (Fig. S4B).

The proportion of physical vs non-physical contacts also differed between reporting methods (${\chi }^{2}$ = 2962, df = 1, p < 0.001), with diary-reported contacts twice as likely to be physical compared with supplementary contacts (Fig. S4C).

### Distance of contacts and participant mobility

**Figure 5:**
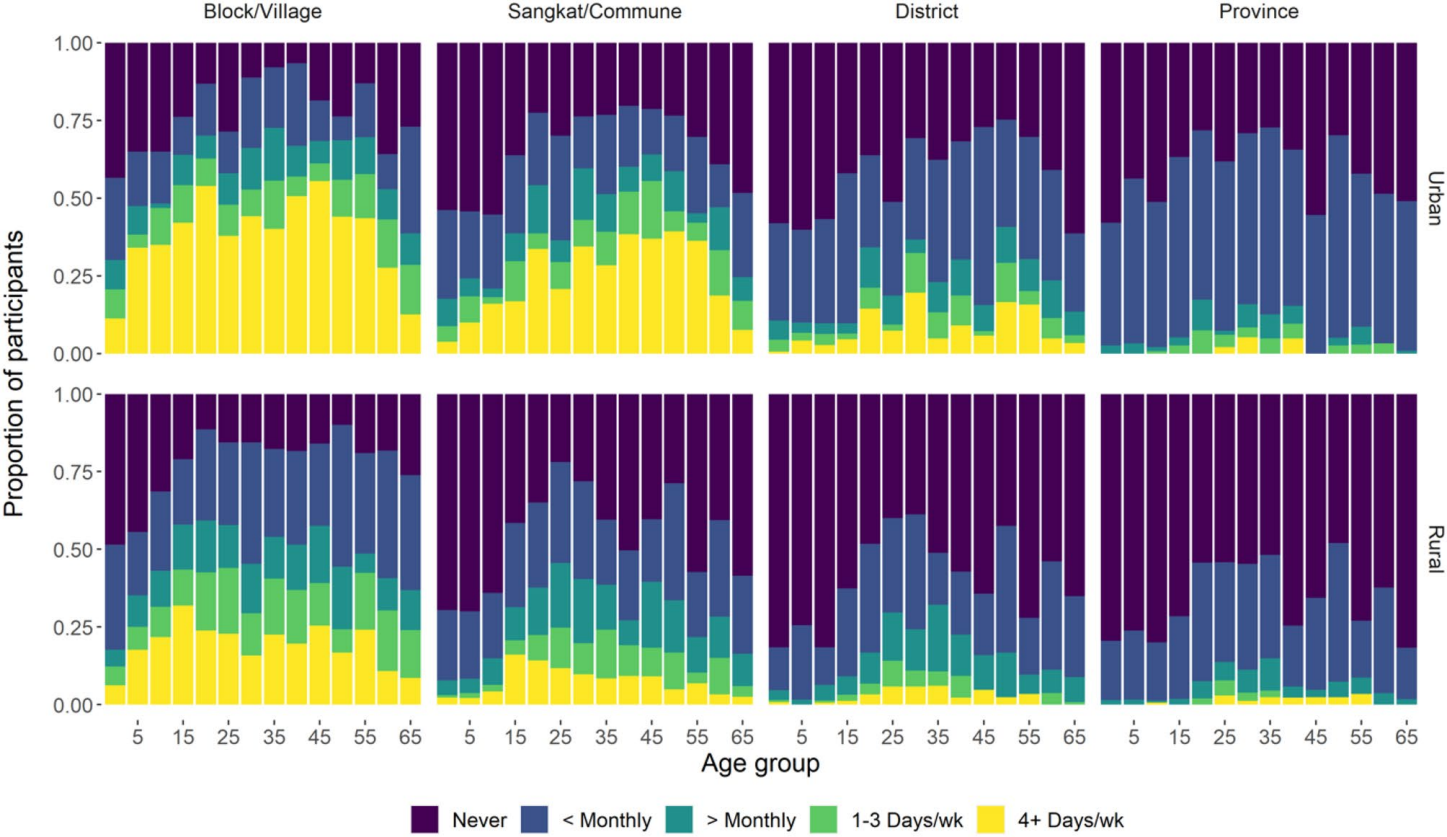
Frequency of travel outside of each administrative division by age group and urban/rural residence.

The majority of diary contacts (88%) occurred within 1 km from participants’ homes and only 2% occurred more than 9 km away from their homes. Based on background questions regarding usual travel frequencies, participants tended to remain fairly localised: 68% and 45% of rural and urban participants, respectively, reported having never left their province, while 98% of rural and 89% of urban residents reported never leaving the country. The oldest and youngest individuals tended to be the least mobile, and rural participants were less mobile than urban participants (Fig. 5).

## Discussion

Here we present a large-scale, prospective social contact patterns study, the first of its kind to be conducted in Cambodia. We estimated that (at least prior to the Covid-19 pandemic) the average Cambodian person had a mean of 31.2 (median: 22; IQR: 13–40) social contacts per day. For comparison to other studies, this figure falls to 16.3 when counting only the contacts that were individually reported in the contact diary. The average number of self-reported diary contacts are similar to the Mossong et al.^13^ European study (13.4 contacts per person per day) and a study in a rural coastal population of Kenya (17.7 contacts per person per day^16^, but higher than other studies in Hong Kong, UK^17^ and Zimbabwe^19^ which reported an average of 7 to 8.1 contacts per person per day. Similar to our study, the study in Zimbabwe also asked participants to list ‘casual’ additional contacts with 64% of people reporting fewer than additional 10 contacts per person per day. Such heterogeneities highlight the need for the collection of context-specific mixing patterns data, although the definition of contact used in the various studies and method of recording contacts will also play a role^18^.

Consistent with studies in other settings, contacts in Cambodia were highly age-assortative; this was particularly evident in school-aged children but was also observed in other age-groups, such as young adults^13,16,28^. Patterns of intergenerational mixing were also evident in our contact matrices. Indeed, when compared with matrices from European countries^13^, intergenerational contacts in Cambodia appeared somewhat more sustained into adulthood, with some of the highest contact rates observed between older (aged > 45 years) and younger people (~ 15–30 years). We also observed notable rates of contact between older generations and young children (e.g. representative of grandparent-grandchild mixing). This likely reflects the higher prevalence of multi-generational households in Cambodia, further supported by our data showing that these contacts tended to be reported at home with household members, particularly in rural areas. This has important implications for respiratory infections such as influenza and SARS-CoV-2, where younger individuals may have higher exposure rates due to their relatively high contact rates, and elderly individuals may be more susceptible severe disease^7^.

Home and school are well documented as high-intensity contact settings important for infectious disease transmission^29,30^. Interventions aiming to reduce contact rates of vulnerable individuals (such as elderly shielding) may be more challenging to implement in LMICs given the household structures. Model simulations indicate that reducing contact rates among younger individuals in LMICs through school closures can have secondary protective effects on older household members^31^. Conversely, concerns have also been raised surrounding potential adverse consequences of such measures, for example if children continue to mix with peers outside of school whilst increasing contact rates with their grandparents, or if there is health care worker absenteeism due to childcare responsibilities^32,33^.

Except professional/office workers, all other occupational groups reported very few contacts at work. Shop workers and traders reported over 75% of their contacts as ‘Home’ either with household or non-household members. Tentatively, this may be indicative of people selling goods from their houses. In future studies, asking participants to specify ‘other’ when collecting contact data would be useful in order to identify important setting-specific locations where contacts may occur. For example, a study in Peru found that a substantial proportion of contacts was made at the market or street which may be relevant here, particularly in rural areas^34^.

Few, if any, previously published contact surveys have differentiated between household and non-household contacts when describing contacts taking place at home (at least in their reported results). Notably, over a quarter of all diary contacts (27.7%) reported in our study occurred at home with non-household members. Given that reduction in rates of inter-household mixing has been a key component in the SARS-CoV-2 pandemic response, this may highlight a need for future contact surveys and modelling studies to consider heterogeneities in contact rates between household vs. non-household members at home. Contacts made in settings outside of home, work or school (defined as “other” settings), also accounted for a substantial proportion of contacts, particularly in rural areas, lending support for the role of NPIs such as public space closures during periods of elevated transmission risk.

A previous study by Prem et al.^7^ combined contact data from the European POLYMOD survey with socio-demographic data with to project social contact matrices for 152 countries, including Cambodia. The overall patterns in our empirical contact matrices were broadly comparable to those in the synthetic, country-level matrices generated by Prem et al.^7^, although we observed a lower relative contribution of contacts in work settings than predicted. Moreover, we observed marked differences between rural and urban areas in terms of the number, intensity and settings of contacts. The higher number of contacts observed in rural areas, particularly in “other” settings, may initially seem counter-intuitive, given higher population densities in urban areas. However, this might be explained by socio-cultural factors such as close-knit rural communities, particularly when considering our survey’s definition of a contact as a two-way conversation. Such factors may also be reflected in higher observed rates of contact made across age groups with visitors at home. Meanwhile, the contacts of urban participants tended to be of higher intensity in terms of both duration and the proportion of contacts which were physical. Heterogeneities within and between countries highlight the importance of the collection of social mixing data from diverse regions^2,21^. However, the contact matrices may serve as valuable inputs for extrapolating to other Southeast Asian countries where such data are not yet available, after appropriate adjustments have been made. Indeed, future studies, which are specifically designed to assess the validity of extrapolating contact survey data to other countries and regions, would be useful to inform considerations and limitations associated with such approaches.

Relatively few previous studies have investigated differences in contact patterns between urban and rural populations. However, our findings are consistent with a study in Kenya, which reported higher contact rates in rural vs peri-urban participants^16^, while studies in Zimbabwe^35^ and China^20^ did not observe any significant differences. Recognizing that early outbreaks of SARS-CoV-2 were mostly observed in cities with subsequent spread to rural areas, Prem et al., (2021) recently updated their synthetic contact matrices to include custom contact matrices for rural and urban settings.

The data analysed in our study were collected over 12 days in May 2012, therefore temporal changes are not captured and changes in social mixing patterns may have occurred since that time, particularly following the emergence of the SARS-CoV-2 pandemic. However, given the lack of data from this region, and LMICs in general^2^, such data are still highly valuable. The POLYMOD data collected in 2005/2006, and “synthetic” contact matrices based on these data, continue to be widely used in infectious disease transmission models, including those developed for SARS-CoV-2^6,9,36,37^. Statistical approaches can be used to adjust for changing population demographics, and our results can also be used to inform and compare with future surveys to identify temporal trends.

Selection bias may have occurred as we observed lower employment rates in our survey participants than has been reported in country census data as a whole. This is likely due to the recruitment via household visits, and future studies recruiting in a similar way may seek to avoid this during collection by having sampling quotas for this variable. We sought to correct for this in our analyses by using sampling weights for employment status (along with other factors such as weekday vs weekend). Such weighting is useful, but care should be taken to avoid raking with too many variables with many levels, and ensuring sufficient data points with each cross-tabulation of these selected variables. This should help to avoid extreme sampling weights and minimise design effects^38^.

Our study used paper-based contact diaries for self-reporting of contacts; other studies have used online surveys, retrospective interviews, or made inferences regarding contacts through wearable devices^39^. Leung et al.^18^ found that those with more years of education and higher income levels were more likely to choose online questionnaires, as opposed to paper based, when both were offered in a study in Hong Kong. They also found that participants using paper questionnaires reported a significantly greater number and duration of contact than those using online questionnaires^18^. Cambodia is classified as an LMIC and, according to the 2010 Demographic Health Survey (DHS), only 19% of rural residents had electricity (compared to 90% of urban residents), necessitating the use of household visits and paper-based questionnaires to reduce selection bias.

Prospective surveys are preferred as they allow participants to remember more details of the contacts^2,40^. The use of an interviewer to retrospectively review and/or complete the contact diaries with participants through prompts enhanced completeness, and also inclusion, for example of illiterate participants (estimated at 30% of females in rural areas in 2012)^41^. However, while participants were asked to report all contacts within the diary, it is worth noting that less than half of our study participants believed their diaries to be complete when asked about this at the end of the interview (participants were not given prior notice that this question would be asked). More contacts were recorded in the diaries (median: 13) compared to supplementary (median: 7). It is difficult to compare these results with other settings as few previous studies have allowed reporting of additional contacts in this manner. Two previous studies did allow reporting of additional ‘casual contacts’ which did not meet the definition of a contact^28,42^. In Uganda, 87% of participants reported casual contacts and 30% of these reported 10 or more casual contacts^28^. In this study, participants reported a median of 7.2 non-casual contacts per person (range 0–25). In South Africa, a median of 14 (IQR: 13–30) casual contacts were reported per person vs. 20 (IQR: 13–29) close contacts^42^. It seems likely that many of the ‘supplementary’ (non-diary) contacts reported in this study would have been more casual in nature, and thus of lower epidemiological relevance. Indeed, a larger proportion of these supplementary contacts were non-physical contacts compared to contact-diary reported contacts. Furthermore, the age distributions of supplementary contacts were reasonably similar to those of the diary contacts (to the extent that these could be compared), suggesting that truncation of the latter would not have biased the age-contact matrices to any substantial degree.

The contact matrices (Table S3) and other data presented here can be used to inform mathematical models of disease transmission in Cambodia. Indeed, the results from this survey were recently used to parameterise an age-structured Susceptible-Exposed-Infectious-Removed (SEIR) model of SARS-CoV-2, to conduct a scenario analysis of NPI strategies on transmission and healthcare resource capacities at country and province-level^43^, under work conducted for the WHO Western-Pacific Regional Office Covid-19 modelling consortium. More broadly, the study also contributes towards addressing knowledge gaps on social-mixing patterns and population mobility in LMIC and Southeast Asian contexts, and provides empirical evidence on within-country variation between urban and rural areas.

## Supplementary Information

Supplementary Information.

## Data Availability

Social Contact Matrices are included in the Supplementary Information and published online (10.5281/zenodo.7642648). Contact data used to generate these matrices are also available online.

## References

[1] Grassly NC, Fraser C (2008). Mathematical models of infectious disease transmission. Nat. Rev. Microbiol..

[2] Hoang T (2019). A systematic review of social contact surveys to inform transmission models of close-contact infections. Epidemiol. Camb. Mass.

[3] Wallinga J, Teunis P, Kretzschmar M (2006). Using data on social contacts to estimate age-specific transmission parameters for respiratory-spread infectious agents. Am. J. Epidemiol..

[4] Eames KTD, Tilston NL, Brooks-Pollock E, Edmunds WJ (2012). Measured dynamic social contact patterns explain the spread of H1N1v influenza. PLOS Comput. Biol..

[5] Kretzschmar M, Teunis PFM, Pebody RG (2010). Incidence and reproduction numbers of Pertussis: Estimates from serological and social contact data in five European countries. PLOS Med..

[6] Prem K (2021). Projecting contact matrices in 177 geographical regions: An update and comparison with empirical data for the COVID-19 era. PLOS Comput. Biol..

[7] Prem K, Cook AR, Jit M (2017). Projecting social contact matrices in 152 countries using contact surveys and demographic data. PLOS Comput. Biol..

[8] Brooks-Pollock E (2021). High COVID-19 transmission potential associated with re-opening universities can be mitigated with layered interventions. Nat. Commun..

[9] Davies NG (2020). Effects of non-pharmaceutical interventions on COVID-19 cases, deaths, and demand for hospital services in the UK: A modelling study. Lancet Public Health.

[10] Head JR (2021). School closures reduced social mixing of children during COVID-19 with implications for transmission risk and school reopening policies. J. R. Soc. Interface.

[11] Prem K (2020). The effect of control strategies to reduce social mixing on outcomes of the COVID-19 epidemic in Wuhan, China: A modelling study. Lancet Public Health.

[12] Zhang J (2020). Changes in contact patterns shape the dynamics of the COVID-19 outbreak in China. Science.

[13] Mossong J (2008). Social contacts and mixing patterns relevant to the spread of infectious diseases. PLOS Med..

[14] Fu Y, Wang D-W, Chuang J-H (2012). Representative contact diaries for modeling the spread of infectious diseases in Taiwan. PLoS ONE.

[15] Horby P (2011). Social contact patterns in vietnam and implications for the control of infectious diseases. PLoS ONE.

[16] Kiti MC (2014). Quantifying age-related rates of social contact using diaries in a rural coastal population of Kenya. PLoS ONE.

[17] Klepac P (2020). Contacts in context: Large-scale setting-specific social mixing matrices from the BBC Pandemic project. MedRxiv.

[18] Leung K, Jit M, Lau EHY, Wu JT (2017). Social contact patterns relevant to the spread of respiratory infectious diseases in Hong Kong. Sci. Rep..

[19] Melegaro A (2017). Social contact structures and time use patterns in the Manicaland Province of Zimbabwe. PLoS ONE.

[20] Read JM (2014). Social mixing patterns in rural and urban areas of southern China. Proc. R. Soc. B Biol. Sci..

[21] Horby P (2012). The epidemiology of interpandemic and pandemic influenza in Vietnam, 2007–2010: The Ha Nam household cohort study I. Am. J. Epidemiol..

[22] Coker RJ, Hunter BM, Rudge JW, Liverani M, Hanvoravongchai P (2011). Emerging infectious diseases in southeast Asia: Regional challenges to control. Lancet.

[23] R Core Team. R: A language and environment for statistical computing. (2020).

[24] Pasek, J. & Pasek, M. J. Package ‘anesrake’. *Compr. R Arch. Netw.* (2018).

[25] Fu Y, Wang D-W, Chuang J-H (2012). Representative contact diaries for modeling the spread of infectious diseases in Taiwan. PLoS ONE.

[26] Funk, S. Socialmixr: social mixing matrices for infectious disease modelling. R package version 0.1.6., (2020).

[27] Rao JNK, Scott AJ (1984). On chi-squared tests for multiway contingency tables with cell proportions estimated from survey data. Ann. Stat..

[28] Le Polain de Waroux, O. *et al.* Characteristics of human encounters and social mixing patterns relevant to infectious diseases spread by close contact: a survey in Southwest Uganda. *BMC Infect. Dis.* **18**, 172 (2018). 10.1186/s12879-018-3073-1 PMC5896105 29642869

[29] Chao DL, Halloran ME, Longini IM (2010). School opening dates predict pandemic influenza A (H1N1) outbreaks in the United States. J. Infect. Dis..

[30] Riley S (2007). Large-scale spatial-transmission models of infectious disease. Science.

[31] Alon, T., Kim, M., Lagakos, D. & VanVuren, M. *How should policy responses to the COVID-19 pandemic differ in the developing world?* (2020).

[32] Bayham J, Fenichel EP (2020). Impact of school closures for COVID-19 on the US health-care workforce and net mortality: A modelling study. Lancet Public Health.

[33] Brooks SK (2020). The impact of unplanned school closure on children’s social contact: Rapid evidence review. EuroSurveillance.

[34] Grijalva CG (2015). A household-based study of contact networks relevant for the spread of infectious diseases in the highlands of Peru. PLoS ONE.

[35] Melegaro A, Jit M, Gay N, Zagheni E, Edmunds WJ (2011). What types of contacts are important for the spread of infections? Using contact survey data to explore European mixing patterns. Epidemics.

[36] Davies NG (2020). Age-dependent effects in the transmission and control of COVID-19 epidemics. Nat. Med..

[37] van Zandvoort K (2020). Response strategies for COVID-19 epidemics in African settings: A mathematical modelling study. BMC Med..

[38] Battaglia MP, Hoaglin DC, Frankel MR (2009). Practical considerations in raking survey data. Surv. Pract..

[39] Read JM, Edmunds WJ, Riley S, Lessler J, Cummings DAT (2012). Close encounters of the infectious kind: Methods to measure social mixing behaviour. Epidemiol. Infect..

[40] Mikolajczyk R, Akmatov M, Rastin S, Kretzschmar M (2008). Social contacts of school children and the transmission of respiratory-spread pathogens. Epidemiol. Infect..

[41] CSES. Cambodian Socioeconomic Health Survey. Cambodia Socio Economic Survey Tables (nis.gov.kh) (2012).

[42] Johnstone-Robertson SP (2011). Social mixing patterns within a South African township community: Implications for respiratory disease transmission and control. Am. J. Epidemiol..

[43] Hidano, A. *et al.* Modelling the impact of non-pharmaceutical interventions on COVID-19 transmission and healthcare demands in Cambodia: a scenario analysis. Technical Report for WHO-WPRO. (2020).

