# Supplementary material for: Social contact patterns relevant for infectious disease transmission in Cambodia
Source: Sci Rep. 2023 Apr 4;13:5542. doi: 10.1038/s41598-023-31485-z (PMC10072808; doi:10.1038/s41598-023-31485-z)
Supplement: Supplementary file 1 — Supplementary Information. [file 41598_2023_31485_MOESM1_ESM.docx]

# Supplementary Information

Table S1. Comparison of the distribution of participant variables with population levels. Data sources for population levels are as follows – urban/rural, sex, age group, number of rooms, employment status from the Cambodian Socioeconomic Survey 2013 (CSES; National Institute of Statistics, 2013), household size from the Cambodia Inter-censal Population Survey, 2013 (CIPS, 2013): <https://www.stat.go.jp/info/meetings/cambodia/pdf/c13ana10323.pdf>; Education from CIPS 2013 <https://www.stat.go.jp/info/meetings/cambodia/pdf/ci_fn02.pdf>; † Highest level of education includes over 25-year olds only. ‡ Employment status includes working age people between 15 and 64 years.

| Covariate |  | Survey % | Population % |
| --- | --- | --- | --- |
| Urban/rural | Urban | 50 | 22 |
|  | Rural | 50 | 78 |
| Interview day | Weekday | 85.2 | 71.4 |
|  | Weekend | 14.8 | 28.6 |
| Sex | Male | 49.9 | 48.9 |
|  | Female | 50 | 51.2 |
| Household size | 1 | 0.6 | 3.6 |
|  | 2 | 3.6 | 10.8 |
|  | 3 | 11.8 | 17.9 |
|  | 4 | 21.5 | 23.2 |
|  | 5 | 20 | 19.2 |
|  | 6 | 15.5 | 12.3 |
|  | 7 | 11.6 | 6.6 |
|  | ≥8 | 15.4 | 6.4 |
| Number of rooms | 1 | 57 | 69.6 |
|  | 2 | 23.4 | 22.4 |
|  | 3 | 10.5 | 5.1 |
|  | 4 | 5.5 | 1.8 |
|  | ≥5 | 3.4 | 1 |
| Age group | 0-4 | 11.5 | 8.8 |
|  | 5-9 | 10 | 9.9 |
|  | 10-14 | 11.9 | 10.4 |
|  | 15-19 | 9.7 | 10.5 |
|  | 20-24 | 7.9 | 11.1 |
|  | 25-29 | 6.4 | 8.7 |
|  | 30-34 | 7.4 | 8.2 |
|  | 35-39 | 6.8 | 4.9 |
|  | 40-44 | 4.3 | 5.6 |
|  | 45-49 | 3.3 | 5.2 |
|  | 50-54 | 3.5 | 4.7 |
|  | 55-59 | 3 | 3.5 |
|  | 60-64 | 5.6 | 2.9 |
|  | ≥65 | 8.8 | 5.7 |
| Highest education † | None | 9.4 | 59.1 |
|  | Primary school | 19 | 22 |
|  | Secondary school | 12.3 | 16 |
|  | High school | 6.3 | 2.9 |
|  | University and above | 1.9 |  |
| Employment status^‡^ | Employed | 70.9 | 84.2 |
|  | Non-employed | 29.1 | 15.8 |

Table S2: Participants included in the survey, and comparison of contact reporting methods (contact diary contacts and supplementary contacts). All values are weighted by urban/rural, weekday/weekend, and employment status. *Some missing data and rounding means these do not always sum to 100%. † Highest level of education includes over 25-year olds only. ‡ Employment status is calculated only working age people between 15 and 64 years.

| Covariate |  |  | --------- Mean number of contacts (SE) --------- | | | Mean hours contact (SE) | ---------- Median number of contacts (IQR) --------- | | Median hours contact (IQR) |
| --- | --- | --- | --- | --- | --- | --- | --- | --- | --- |
|  |  | Freq (%) * | Total | Contact diary | Supplementary | Contact diary | Total | Contact diary | Supplementary |
| Overall | - N/A - | 2016 (100) | 30.7 (0.7) | 15.9 (0.3) | 14.8 (0.6) | 30.2 (0.5) | 22 (13-40) | 13 (8-20) | 7 (0-21) |
| Urban/rural | Urban | 1008 (50) | 26.7 (0.9) | 11.7 (0.2) | 15.1 (0.9) | 25.8 (0.6) | 19 (11-36) | 10 (7-15) | 6 (0-21) |
|  | Rural | 1008 (50) | 31.8 (0.8) | 17.1 (0.4) | 14.7 (0.7) | 31.5 (0.7) | 24 (14-41) | 14 (9-22) | 7 (0-21) |
| Day of the week | Weekday | 1717 (85.2) | 32.8 (0.8) | 16.3 (0.3) | 16.4 (0.7) | 31.1 (0.6) | 25 (14-43) | 13 (9-20) | 8 (0-24) |
|  | Weekend | 299 (14.8) | 25.6 (1.3) | 15 (0.7) | 10.6 (1) | 28.2 (1.2) | 20 (12-33) | 12 (8-18) | 5 (0-16) |
| Sex | Male | 1006 (49.9) | 31.1 (1) | 16.1 (0.5) | 15 (0.8) | 30.4 (0.7) | 24 (13-42) | 12 (8-20) | 7 (0-23) |
|  | Female | 1007 (50) | 30.3 (1) | 15.8 (0.4) | 14.5 (0.8) | 30 (0.8) | 22 (13-39) | 13 (9-19) | 6 (0-20) |
| Household size | 1 | 13 (0.6) | 41.8 (12.6) | 13.8 (2.4) | 28.1 (12.6) | 11.8 (4.4) | 28 (19-38) | 10 (9-23) | 9 (0-32) |
| (people) | 2 | 72 (3.6) | 24.5 (2.1) | 14.8 (1.7) | 9.7 (1.6) | 16.1 (1.8) | 19 (13-33) | 10 (6-20) | 4 (0-12) |
|  | 3 | 238 (11.8) | 31.5 (1.7) | 16.9 (1) | 14.6 (1.3) | 26.9 (1.7) | 28 (14-48) | 13 (8-25) | 10 (0-24) |
|  | 4 | 433 (21.5) | 32.5 (1.7) | 16.2 (0.7) | 16.3 (1.4) | 28.3 (1.2) | 22 (14-41) | 13 (9-20) | 8 (0-22) |
|  | 5 | 404 (20) | 30.7 (1.5) | 16.5 (0.8) | 14.2 (1.2) | 31 (1.2) | 20 (12-44) | 12 (8-20) | 6 (0-21) |
|  | 6 | 312 (15.5) | 31.6 (1.9) | 15.7 (0.8) | 15.8 (1.6) | 32.8 (1.4) | 24 (14-40) | 12 (8-18) | 7 (0-21) |
|  | 7 | 234 (11.6) | 29 (1.8) | 15.6 (0.9) | 13.4 (1.4) | 32.9 (1.5) | 22 (12-39) | 12 (9-18) | 5 (0-21) |
|  | ≥8 | 310 (15.4) | 28.6 (1.6) | 14.8 (0.5) | 13.8 (1.4) | 35.6 (1.3) | 21 (13-35) | 12 (10-18) | 6 (0-21) |
| Number of | 1 | 1150 (57) | 31.6 (0.9) | 17.3 (0.4) | 14.3 (0.7) | 31.4 (0.7) | 23 (14-41) | 14 (9-22) | 6 (0-21) |
| rooms | 2 | 472 (23.4) | 29.2 (1.4) | 13.6 (0.6) | 15.6 (1.2) | 28.7 (1.2) | 21 (12-39) | 11 (7-18) | 9 (0-23) |
|  | 3 | 212 (10.5) | 29.1 (2) | 12.9 (0.8) | 16.2 (1.7) | 26.5 (1.3) | 21 (14-38) | 11 (8-16) | 10 (0-23) |
|  | 4 | 111 (5.5) | 25.3 (2.3) | 11.2 (0.7) | 14.1 (2.1) | 26.5 (1.9) | 18 (11-31) | 10 (8-15) | 6 (0-19) |
|  | ≥5 | 69 (3.4) | 29.9 (6.3) | 10.4 (0.7) | 19.5 (6.3) | 25.7 (2.1) | 24 (9-37) | 9 (7-13) | 10 (0-24) |
| Age group | 0-4 | 231 (11.5) | 20.4 (1.1) | 13.7 (0.6) | 6.6 (1.1) | 27.9 (1.2) | 17 (11-27) | 13 (9-17) | 0 (0-9) |
|  | 5-9 | 201 (10) | 25 (1.5) | 17.6 (1) | 7.3 (1.1) | 39.4 (2.2) | 18 (12-33) | 14 (10-24) | 0 (0-10) |
|  | 10-15 | 239 (11.9) | 31.1 (1.6) | 19 (1) | 12.1 (1.3) | 40 (1.9) | 26 (16-40) | 16 (10-24) | 5 (0-19) |
|  | 15-19 | 195 (9.7) | 35.9 (2.3) | 18 (1.1) | 17.9 (1.7) | 35.6 (2) | 28 (15-46) | 14 (10-22) | 13 (0-29) |
|  | 20-24 | 160 (7.9) | 36.3 (2.7) | 17.5 (1.4) | 18.8 (1.9) | 33.4 (2.4) | 25 (15-59) | 13 (8-23) | 13 (0-27) |
|  | 25-29 | 129 (6.4) | 34.4 (3.1) | 17.6 (1.4) | 16.8 (2.9) | 32.7 (2.3) | 25 (13-47) | 12 (9-23) | 6 (0-24) |
|  | 30-34 | 149 (7.4) | 37 (3) | 16.3 (1.2) | 20.7 (2.4) | 26.7 (1.4) | 27 (16-49) | 13 (9-20) | 12 (3-31) |
|  | 35-39 | 137 (6.8) | 36.1 (3) | 15.8 (1.1) | 20.3 (2.5) | 26.6 (1.6) | 25 (15-48) | 14 (9-20) | 12 (0-30) |
|  | 40-44 | 87 (4.3) | 35.6 (3.8) | 13.7 (1.1) | 21.9 (3.1) | 25.1 (1.7) | 23 (13-49) | 10 (8-18) | 12 (3-37) |
|  | 45-49 | 66 (3.3) | 29.3 (3.5) | 15.7 (1.7) | 13.6 (2.9) | 26.7 (2.9) | 21 (10-45) | 11 (8-18) | 6 (0-20) |
|  | 50-54 | 71 (3.5) | 35.4 (3.8) | 16.1 (1.7) | 19.3 (3) | 30.3 (3.2) | 29 (18-40) | 12 (8-20) | 16 (5-24) |
|  | 55-59 | 61 (3) | 29.5 (3.4) | 13.4 (2) | 16.1 (2.4) | 22.1 (1.8) | 26 (13-46) | 11 (6-16) | 12 (2-29) |
|  | 60-64 | 113 (5.6) | 27.6 (2.1) | 13.8 (1) | 13.8 (2) | 24.2 (1.6) | 22 (13-35) | 12 (8-18) | 7 (0-21) |
|  | ≥65 | 177 (8.8) | 20.4 (1.4) | 11.8 (0.8) | 8.7 (1.2) | 21.1 (1.2) | 15 (9-28) | 9 (6-15) | 0 (0-10) |
| Occupation | Child at home | 286 (14.2) | 21.1 (1) | 14.9 (0.7) | 6.2 (0.9) | 30 (1.3) | 17 (12-29) | 13 (9-18) | 0 (0-8) |
|  | Pre-schooler | 75 (3.7) | 31.4 (3) | 20.4 (1.7) | 11 (2) | 46.2 (4.5) | 26 (14-45) | 17 (10-30) | 0 (0-16) |
|  | School/college student | 466 (23.1) | 31.2 (1.4) | 17.2 (0.7) | 14.1 (1.2) | 37.3 (1.3) | 23 (15-40) | 15 (10-21) | 6 (0-21) |
|  | Professional/office worker | 84 (4.2) | 33.4 (3.6) | 12.2 (0.9) | 21.2 (3) | 25.6 (1.9) | 27 (15-45) | 10 (8-15) | 15 (4-27) |
|  | Shop worker/trader | 165 (8.2) | 39.9 (3.4) | 11.8 (0.8) | 28.1 (3) | 22.9 (1.3) | 29 (16-49) | 10 (6-15) | 19 (7-40) |
|  | Manual labourer, non-agri | 131 (6.5) | 30 (3) | 12.2 (1) | 17.8 (2.9) | 25.2 (2) | 22 (11-42) | 10 (7-14) | 7 (0-26) |
|  | Agriculture and fishing | 459 (22.8) | 33.1 (1.3) | 17.9 (0.6) | 15.3 (1) | 30.1 (1) | 25 (14-48) | 14 (9-22) | 10 (0-22) |
|  | Housewife | 134 (6.6) | 22.6 (1.8) | 13.6 (1.1) | 9 (1.3) | 25 (1.3) | 19 (9-30) | 10 (7-19) | 1 (0-15) |
|  | Retired | 54 (2.7) | 22.2 (2.7) | 12 (1.5) | 10.2 (2.3) | 20.1 (2.8) | 16 (9-33) | 11 (8-13) | 3 (0-14) |
|  | Unemployed | 95 (4.7) | 26.1 (2.6) | 13.5 (1.4) | 12.6 (2.2) | 25.2 (2.3) | 19 (11-31) | 9 (7-17) | 5 (0-18) |
|  | Others | 64 (3.2) | 36.6 (3.8) | 15.2 (1) | 21.4 (3.5) | 28.5 (2.7) | 30 (16-45) | 14 (10-21) | 17 (0-29) |
| Highest level of | None | 189 (19.2) | 28.3 (2) | 14.1 (0.9) | 14.2 (1.7) | 25.1 (1.4) | 21 (13-35) | 11 (7-18) | 7 (0-21) |
| education | Primary school | 383 (19.0) | 32.4 (1.7) | 16 (0.7) | 16.5 (1.4) | 27.4 (1.1) | 23 (14-41) | 13 (8-19) | 8 (0-24) |
| completed† | Secondary school | 247 (38.9) | 33.6 (2.1) | 15.4 (1) | 18.2 (1.7) | 26.3 (1.4) | 26 (12-50) | 11 (7-18) | 10 (0-30) |
|  | High school | 128 (13.0) | 28 (2.3) | 12.8 (1.1) | 15.2 (2) | 24.8 (1.4) | 22 (11-36) | 10 (7-16) | 8 (0-25) |
|  | University and above | 38 (3.9) | 43.4 (9.2) | 12 (1.5) | 31.4 (8.8) | 22.9 (2.5) | 25 (18-47) | 9 (7-16) | 15 (6-32) |
| Employment | Employed | 828 (70.9) | 34.5 (1.1) | 16.2 (0.5) | 18.2 (0.9) | 28.7 (0.8) | 26 (15-48) | 12 (8-20) | 11 (0-26) |
| status ‡ | Non-employed | 340 (29.1) | 33.4 (1.9) | 15.4 (0.7) | 18 (1.6) | 30.7 (1.3) | 25 (14-43) | 12 (8-19) | 11 (0-28) |
|  |  |  |  |  |  |  |  |  |  |

Figure S1: Contact matrices for contacts that were either physical in nature or at least 15 minutes in duration. Each cell shows the mean number of contacts made between age groups. All matrices are weighted by day of the week (weekday or weekend) and employment status (non-working age, employed, or unemployed). Matrices were further adjusted to account for recipricocity of contacts such that the total number of contacts that age group (i) made with age group (j), were equal to the total number of contacts age grosup (j) made with age group (i) (c_ij_N_i_ = c_ji_N_j_ ; where N is the population size of an age group).


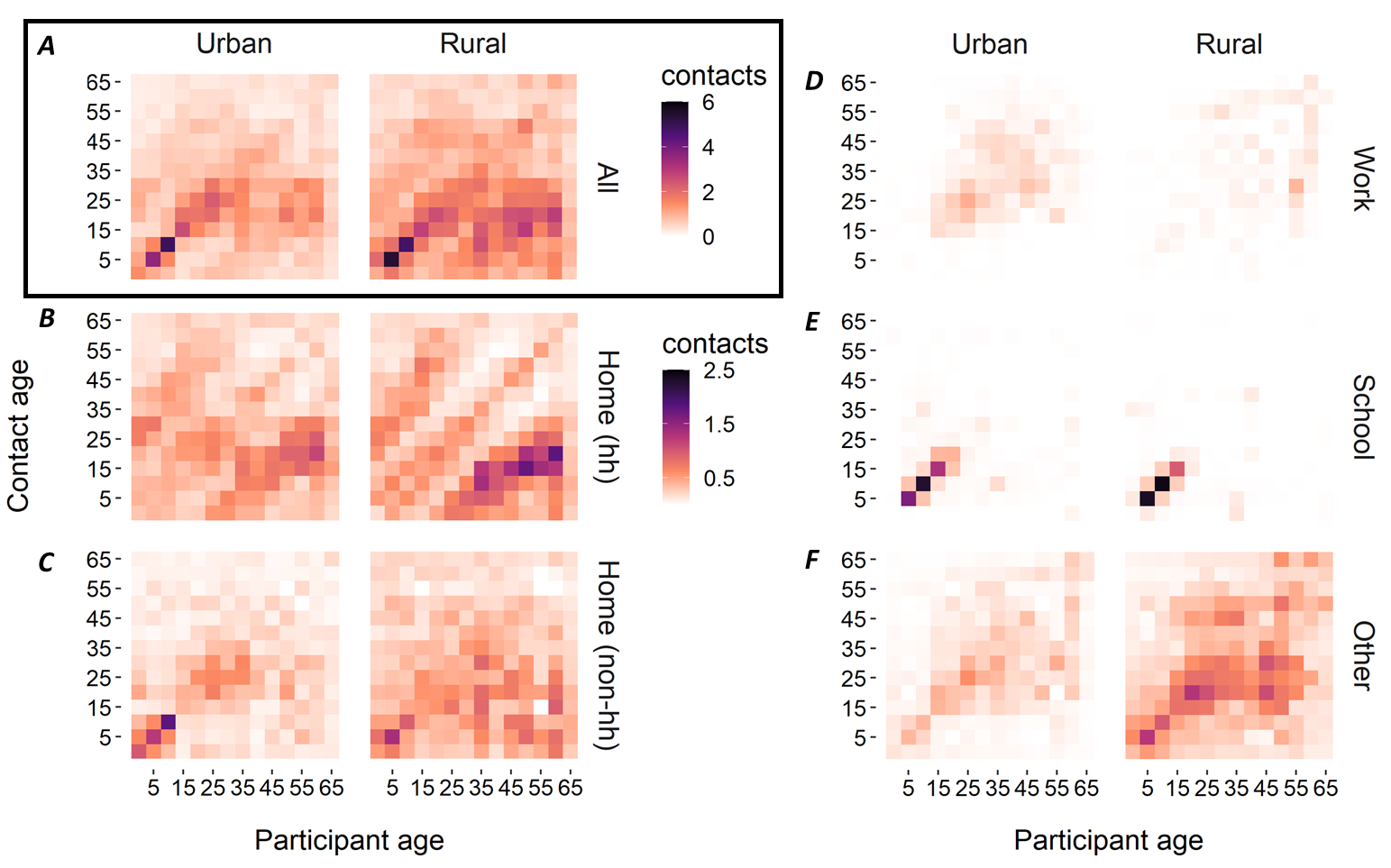


Figure S2: relationship between different measures of contact intensity. A) the proportion of physical contacts by duration that the contacts lasted; B) the proportion of physical contacts according to the frequency that contacts took place; C) the proportion of contacts lasting each duration, according to the frequency that the contact took place.


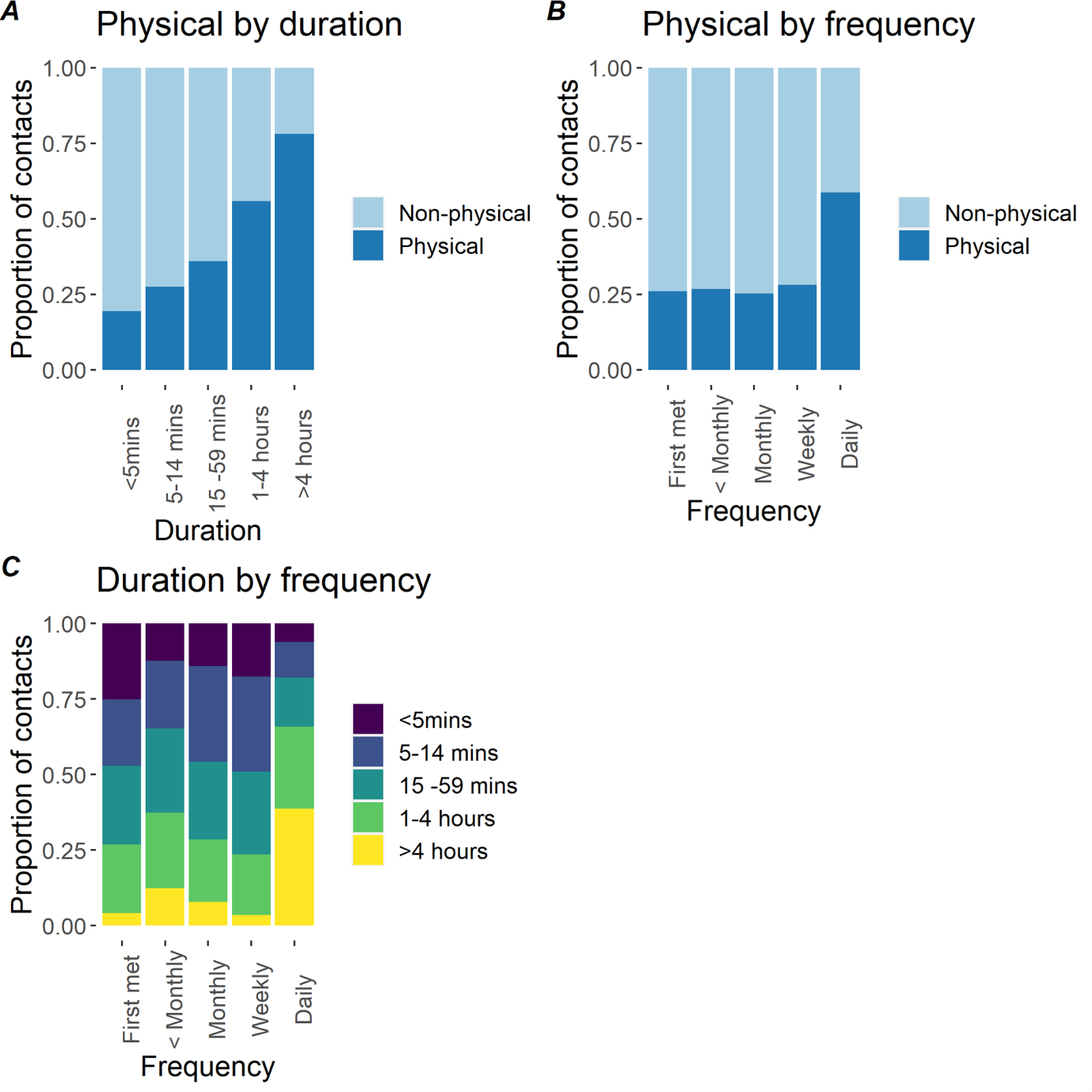


Figure S3: Degree distribution (number of contacts recorded) according to different reporting methods – contact diary contacts, supplementary contacts, and overall.


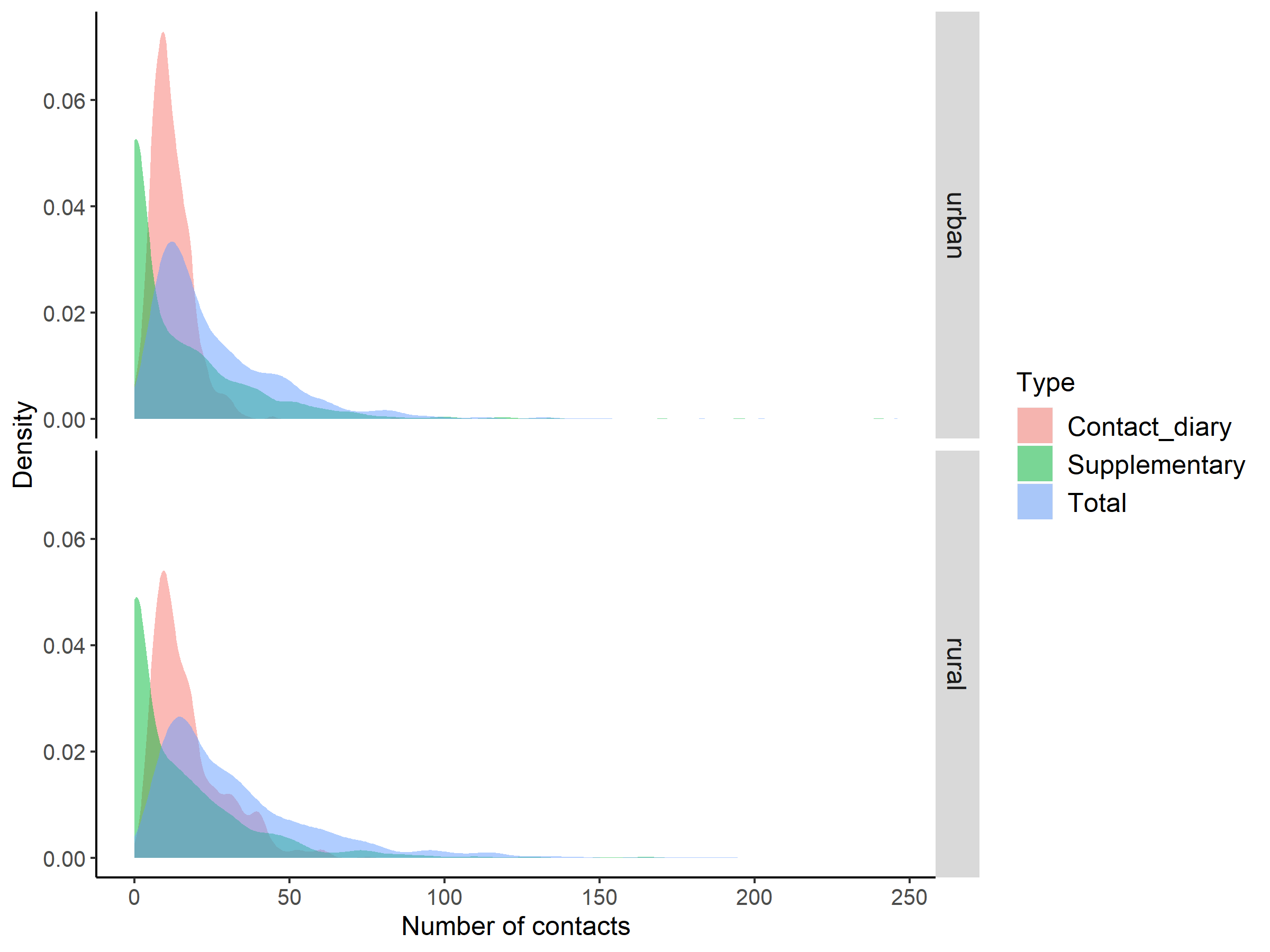


Figure S4: Comparison of the types of contacts reported the contact diary (CD) vs as supplementary contacts (Supp). (A) age distributions of contacts (in years) by each reporting type, (B) age-structured mixing matrices for supplementary contacts, and contact diary contacts coerced into the same age bands, (C) proportion of physical contacts by each reporting type.


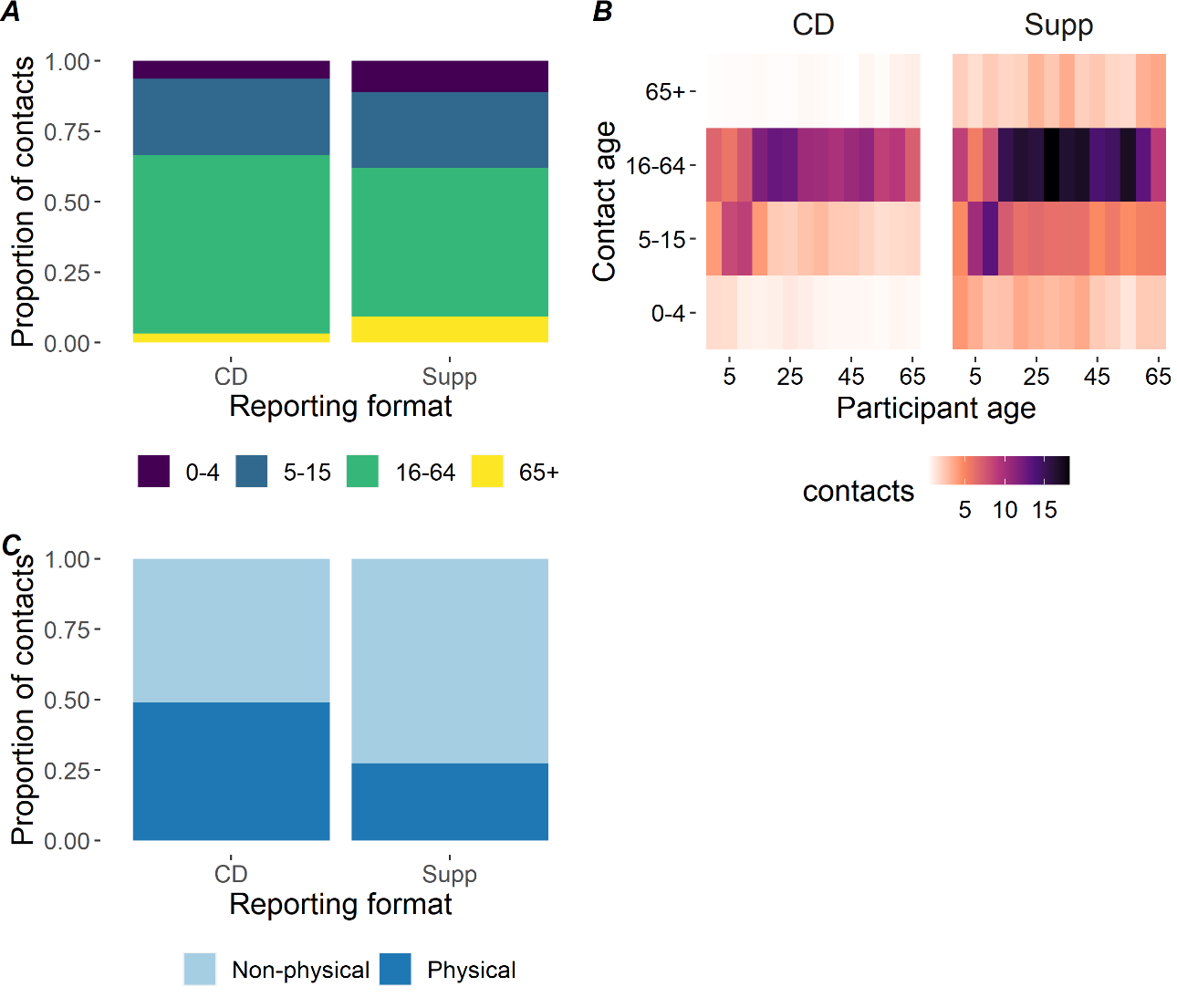


Figure S5: Demographic structure of Cambodia by urban and rural. Data source: Cambodian Socioeconomic Survey 2013 (CSES; National Institute of Statistics, 2013)


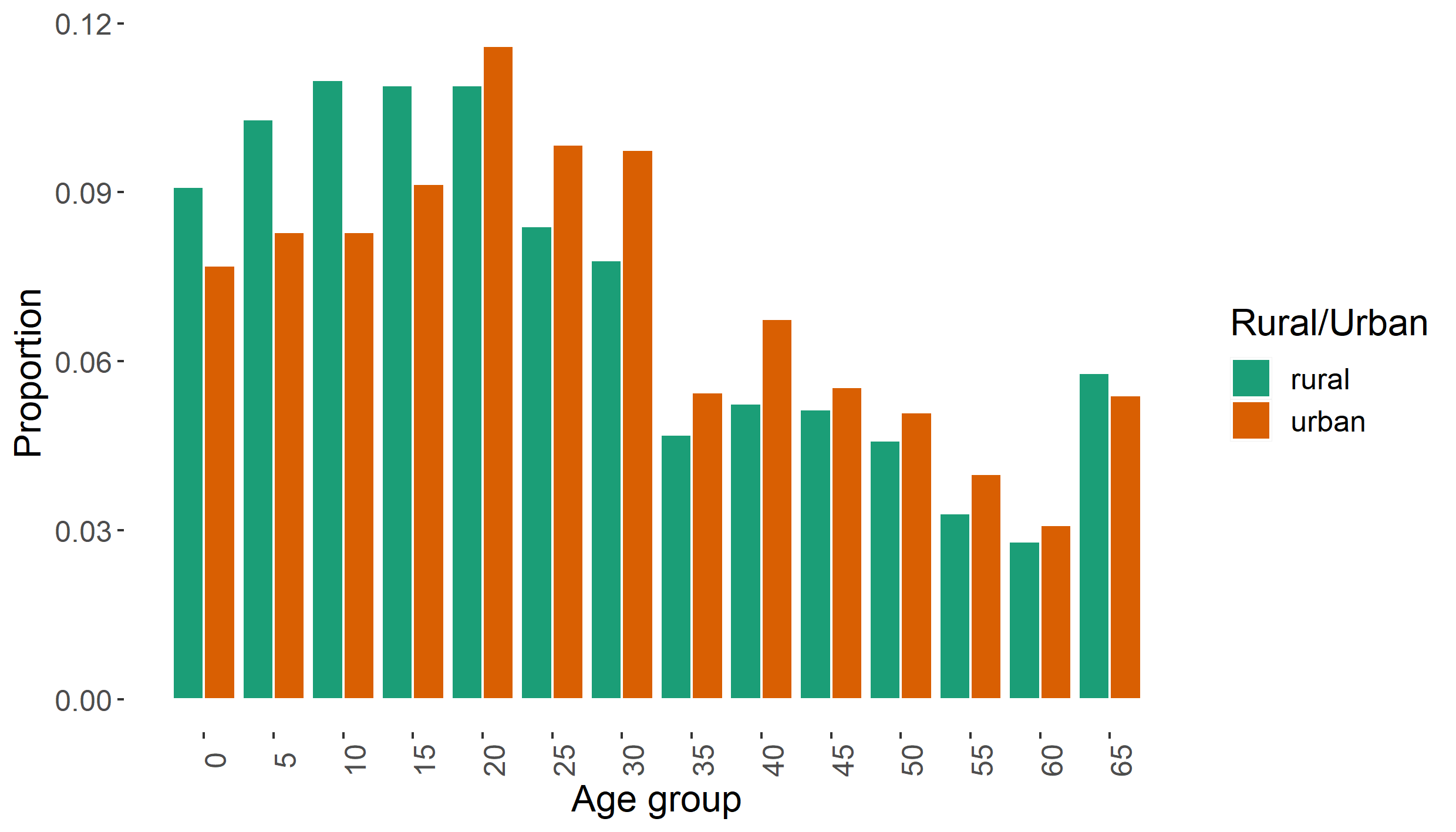


*Table S3 Social contact matrices generated from the study according to urban/rural location and setting*

All; urban

|  | 0 | 5 | 10 | 15 | 20 | 25 | 30 | 35 | 40 | 45 | 50 | 55 | 60 | 65 |
| --- | --- | --- | --- | --- | --- | --- | --- | --- | --- | --- | --- | --- | --- | --- |
| 0 | 1.439139 | 1.072134 | 0.791639 | 0.416281 | 0.67856 | 0.875934 | 0.782377 | 0.660771 | 0.558637 | 0.414646 | 0.491517 | 0.425567 | 0.506165 | 0.34909 |
| 5 | 1.15568 | 3.634464 | 1.561204 | 0.453524 | 0.43344 | 0.555795 | 0.836017 | 0.837733 | 0.723019 | 0.631261 | 0.369837 | 0.746398 | 0.771912 | 0.409593 |
| 10 | 0.853328 | 1.561204 | 5.172298 | 0.878524 | 0.723236 | 0.552648 | 0.569083 | 1.307411 | 1.021305 | 1.020034 | 0.738215 | 0.55513 | 0.797273 | 0.505866 |
| 15 | 0.494672 | 0.499968 | 0.968491 | 2.872554 | 1.729223 | 1.330142 | 0.944981 | 1.523381 | 1.082732 | 1.07145 | 1.238402 | 1.517585 | 1.234213 | 0.796611 |
| 20 | 1.022248 | 0.605772 | 1.010787 | 2.192245 | 2.131972 | 2.5686 | 1.412569 | 1.95165 | 1.662105 | 1.489148 | 2.312643 | 2.184879 | 2.138674 | 0.736039 |
| 25 | 1.120515 | 0.659588 | 0.655853 | 1.431904 | 2.181094 | 2.983449 | 2.29104 | 2.030917 | 1.23034 | 1.010828 | 1.741204 | 1.698939 | 1.907609 | 0.71267 |
| 30 | 0.990675 | 0.982068 | 0.668501 | 1.006949 | 1.187289 | 2.267782 | 1.611235 | 2.204329 | 1.83052 | 1.440901 | 1.53798 | 2.130768 | 2.037278 | 0.755421 |
| 35 | 0.46769 | 0.550078 | 0.858482 | 0.907372 | 0.916941 | 1.123708 | 1.232165 | 1.410594 | 1.15609 | 0.872989 | 0.896424 | 0.645252 | 0.710275 | 0.427589 |
| 40 | 0.489714 | 0.587996 | 0.830577 | 0.798737 | 0.96717 | 0.843125 | 1.26728 | 1.431849 | 1.701227 | 1.103242 | 0.757139 | 0.675326 | 0.996026 | 0.716482 |
| 45 | 0.298869 | 0.422108 | 0.682071 | 0.649898 | 0.71248 | 0.569553 | 0.820205 | 0.889006 | 0.907113 | 1.325946 | 0.614345 | 0.65944 | 0.706487 | 0.340371 |
| 50 | 0.325551 | 0.227249 | 0.453602 | 0.690258 | 1.016766 | 0.901538 | 0.804482 | 0.838854 | 0.572062 | 0.564533 | 0.873863 | 0.479401 | 0.68675 | 0.386918 |
| 55 | 0.221075 | 0.359711 | 0.267533 | 0.663429 | 0.753409 | 0.689927 | 0.874164 | 0.47358 | 0.400196 | 0.475274 | 0.376002 | 0.448864 | 0.688515 | 0.24383 |
| 60 | 0.203782 | 0.288305 | 0.297778 | 0.418151 | 0.571544 | 0.600367 | 0.647752 | 0.404011 | 0.457437 | 0.394616 | 0.417438 | 0.5336 | 0.487185 | 0.341306 |
| 65 | 0.244816 | 0.266481 | 0.329116 | 0.470131 | 0.342638 | 0.390701 | 0.418386 | 0.423664 | 0.573185 | 0.33117 | 0.409676 | 0.329168 | 0.594529 | 0.666403 |

All; rural

|  | 0 | 5 | 10 | 15 | 20 | 25 | 30 | 35 | 40 | 45 | 50 | 55 | 60 | 65 |
| --- | --- | --- | --- | --- | --- | --- | --- | --- | --- | --- | --- | --- | --- | --- |
| 0 | 1.157491 | 1.996312 | 1.257754 | 0.883692 | 1.044741 | 1.319355 | 1.214381 | 1.384626 | 0.917801 | 1.21925 | 0.837725 | 1.027973 | 1.887139 | 0.6403 |
| 5 | 2.259563 | 5.850188 | 2.026918 | 1.205719 | 1.168641 | 1.483443 | 1.745745 | 2.359374 | 1.52176 | 1.371797 | 1.727524 | 1.598452 | 1.93089 | 1.049628 |
| 10 | 1.520363 | 2.164671 | 5.353858 | 2.232948 | 1.357921 | 1.03093 | 2.196982 | 3.135237 | 1.964027 | 2.581266 | 2.064659 | 1.616232 | 1.94696 | 1.284226 |
| 15 | 1.058489 | 1.275955 | 2.212647 | 3.425259 | 2.423567 | 2.357116 | 1.889601 | 3.37246 | 2.214461 | 2.897685 | 4.015185 | 2.90471 | 3.085519 | 0.974744 |
| 20 | 1.251393 | 1.236717 | 1.345576 | 2.423567 | 3.177525 | 2.786842 | 2.495267 | 2.884303 | 2.727059 | 3.280452 | 3.180278 | 3.035677 | 4.270653 | 1.178787 |
| 25 | 1.217867 | 1.209798 | 0.787255 | 1.816494 | 2.147659 | 2.626798 | 2.503029 | 2.382431 | 1.734604 | 2.804157 | 2.249611 | 3.685995 | 2.832198 | 1.021266 |
| 30 | 1.040898 | 1.32202 | 1.557859 | 1.352191 | 1.785603 | 2.32424 | 2.614413 | 3.069298 | 2.012095 | 2.421224 | 2.349985 | 3.364352 | 2.598697 | 1.238961 |
| 35 | 0.715137 | 1.076608 | 1.339601 | 1.454181 | 1.243691 | 1.333027 | 1.84945 | 2.01587 | 1.39398 | 1.562306 | 1.32046 | 1.779197 | 1.258183 | 0.779704 |
| 40 | 0.529501 | 0.775654 | 0.937376 | 1.066598 | 1.313492 | 1.084128 | 1.354296 | 1.557105 | 1.500952 | 1.346678 | 1.444381 | 1.095362 | 1.534652 | 0.557076 |
| 45 | 0.690015 | 0.685898 | 1.2085 | 1.369089 | 1.549938 | 1.719213 | 1.598628 | 1.711886 | 1.321026 | 1.548887 | 1.673179 | 1.315453 | 1.247699 | 0.639143 |
| 50 | 0.423465 | 0.771515 | 0.863402 | 1.694481 | 1.342135 | 1.231928 | 1.385889 | 1.292364 | 1.265552 | 1.49449 | 1.900435 | 1.106322 | 1.154333 | 0.827099 |
| 55 | 0.372781 | 0.512125 | 0.484869 | 0.879407 | 0.919057 | 1.448067 | 1.423379 | 1.249221 | 0.688512 | 0.842911 | 0.793665 | 1.438807 | 0.738678 | 0.461742 |
| 60 | 0.580659 | 0.524903 | 0.49559 | 0.792611 | 1.09705 | 0.944067 | 0.932867 | 0.749556 | 0.818482 | 0.678362 | 0.702639 | 0.626759 | 0.876213 | 0.629726 |
| 65 | 0.408104 | 0.591053 | 0.677137 | 0.518672 | 0.627245 | 0.70516 | 0.92128 | 0.962187 | 0.615437 | 0.719812 | 1.042864 | 0.811548 | 1.304431 | 0.944834 |

Home with household members; urban

|  | 0 | 5 | 10 | 15 | 20 | 25 | 30 | 35 | 40 | 45 | 50 | 55 | 60 | 65 |
| --- | --- | --- | --- | --- | --- | --- | --- | --- | --- | --- | --- | --- | --- | --- |
| 0 | 0.301475 | 0.374264 | 0.339666 | 0.23975 | 0.284727 | 0.558634 | 0.567195 | 0.404466 | 0.406406 | 0.23298 | 0.280792 | 0.285894 | 0.33895 | 0.216341 |
| 5 | 0.403428 | 0.357532 | 0.384474 | 0.229205 | 0.195057 | 0.370287 | 0.640997 | 0.530056 | 0.525899 | 0.353899 | 0.246694 | 0.280743 | 0.355995 | 0.270792 |
| 10 | 0.366135 | 0.384474 | 0.426284 | 0.382058 | 0.327001 | 0.267963 | 0.290839 | 0.713864 | 0.708288 | 0.772568 | 0.407492 | 0.188551 | 0.483468 | 0.360236 |
| 15 | 0.284898 | 0.252677 | 0.421184 | 0.336872 | 0.443304 | 0.558624 | 0.325951 | 0.77559 | 0.597932 | 0.806905 | 0.752297 | 0.881506 | 0.852924 | 0.571689 |
| 20 | 0.42894 | 0.272609 | 0.457014 | 0.562004 | 0.548439 | 0.692853 | 0.452699 | 0.717391 | 0.546874 | 0.818906 | 0.938824 | 0.90732 | 1.319491 | 0.495676 |
| 25 | 0.714618 | 0.439437 | 0.318004 | 0.601361 | 0.588328 | 0.719694 | 0.559824 | 0.364121 | 0.261886 | 0.266653 | 0.833121 | 0.875374 | 1.010403 | 0.389927 |
| 30 | 0.718203 | 0.752978 | 0.341648 | 0.347325 | 0.380501 | 0.554141 | 0.419413 | 0.314633 | 0.392807 | 0.333822 | 0.443673 | 0.759841 | 0.788155 | 0.447301 |
| 35 | 0.286279 | 0.348049 | 0.468743 | 0.461965 | 0.337051 | 0.201468 | 0.175872 | 0.324579 | 0.31403 | 0.12788 | 0.180167 | 0.143067 | 0.253158 | 0.179528 |
| 40 | 0.356265 | 0.427688 | 0.576015 | 0.441097 | 0.318224 | 0.179465 | 0.271943 | 0.388934 | 0.551244 | 0.266686 | 0.202771 | 0.063224 | 0.219543 | 0.436058 |
| 45 | 0.167927 | 0.236643 | 0.516596 | 0.489436 | 0.391804 | 0.150246 | 0.190022 | 0.130226 | 0.219276 | 0.425133 | 0.163277 | 0.080091 | 0.128293 | 0.26019 |
| 50 | 0.18598 | 0.151583 | 0.250386 | 0.419314 | 0.412759 | 0.431362 | 0.232075 | 0.168596 | 0.153206 | 0.150039 | 0.349661 | 0.170654 | 0.132598 | 0.246319 |
| 55 | 0.148517 | 0.135298 | 0.090868 | 0.38536 | 0.31287 | 0.355483 | 0.31173 | 0.105004 | 0.037466 | 0.057724 | 0.133847 | 0.090912 | 0.324487 | 0.112499 |
| 60 | 0.136461 | 0.132962 | 0.180573 | 0.28897 | 0.352624 | 0.317996 | 0.250594 | 0.143998 | 0.100828 | 0.071659 | 0.080599 | 0.251478 | 0.174183 | 0.10399 |
| 65 | 0.15172 | 0.176177 | 0.234369 | 0.33739 | 0.230745 | 0.213767 | 0.247735 | 0.17788 | 0.348846 | 0.253157 | 0.260808 | 0.151872 | 0.181142 | 0.21638 |

Home with household members; rural

|  | 0 | 5 | 10 | 15 | 20 | 25 | 30 | 35 | 40 | 45 | 50 | 55 | 60 | 65 |
| --- | --- | --- | --- | --- | --- | --- | --- | --- | --- | --- | --- | --- | --- | --- |
| 0 | 0.178787 | 0.482577 | 0.296396 | 0.246108 | 0.46444 | 0.760084 | 0.735833 | 0.582803 | 0.497413 | 0.538399 | 0.342209 | 0.339251 | 0.706226 | 0.241267 |
| 5 | 0.546214 | 0.461512 | 0.592776 | 0.368614 | 0.230179 | 0.666181 | 0.901649 | 0.970494 | 0.876132 | 0.514431 | 0.239265 | 0.392696 | 0.684557 | 0.341262 |
| 10 | 0.358281 | 0.633062 | 0.351866 | 0.585401 | 0.328239 | 0.225079 | 0.635368 | 1.363742 | 1.00748 | 0.885397 | 0.599707 | 0.533373 | 0.761924 | 0.40791 |
| 15 | 0.294789 | 0.390086 | 0.580079 | 0.498111 | 0.562395 | 0.391151 | 0.280788 | 1.241483 | 1.041539 | 1.381042 | 1.806786 | 1.452416 | 1.300462 | 0.307272 |
| 20 | 0.556308 | 0.243587 | 0.325255 | 0.562395 | 0.698984 | 0.523821 | 0.245576 | 0.29117 | 0.712932 | 0.700002 | 1.366375 | 1.378889 | 1.846535 | 0.298585 |
| 25 | 0.701616 | 0.543294 | 0.171879 | 0.301438 | 0.403679 | 0.529229 | 0.514054 | 0.239011 | 0.190691 | 0.334098 | 0.58556 | 0.972087 | 0.837158 | 0.211635 |
| 30 | 0.630714 | 0.682802 | 0.450533 | 0.200931 | 0.175733 | 0.477336 | 0.644593 | 0.378679 | 0.200858 | 0.094851 | 0.252903 | 0.587793 | 0.560084 | 0.299298 |
| 35 | 0.301008 | 0.442847 | 0.58269 | 0.535319 | 0.12555 | 0.133732 | 0.228179 | 0.497857 | 0.202741 | 0.079479 | 0.037973 | 0.139254 | 0.227753 | 0.236622 |
| 40 | 0.286969 | 0.446572 | 0.480843 | 0.501659 | 0.343385 | 0.119182 | 0.135193 | 0.226466 | 0.390198 | 0.181056 | 0.192904 | 0.012278 | 0.165867 | 0.105456 |
| 45 | 0.304698 | 0.257215 | 0.414526 | 0.65251 | 0.330735 | 0.204834 | 0.062626 | 0.087088 | 0.177607 | 0.56683 | 0.362962 | 0.063242 | 0.088129 | 0.09478 |
| 50 | 0.172985 | 0.106856 | 0.250786 | 0.762497 | 0.576635 | 0.320664 | 0.149148 | 0.037165 | 0.16902 | 0.324199 | 0.529867 | 0.159676 | 0.190771 | 0.100359 |
| 55 | 0.123025 | 0.125815 | 0.160012 | 0.439722 | 0.417461 | 0.381891 | 0.248682 | 0.097774 | 0.007718 | 0.040524 | 0.11455 | 0.488717 | 0.203902 | 0.108834 |
| 60 | 0.217301 | 0.186093 | 0.193945 | 0.334064 | 0.47434 | 0.279053 | 0.201056 | 0.135683 | 0.088463 | 0.047915 | 0.116122 | 0.173008 | 0.30053 | 0.081806 |
| 65 | 0.153775 | 0.192167 | 0.21508 | 0.163503 | 0.15888 | 0.146129 | 0.222555 | 0.292002 | 0.116504 | 0.106742 | 0.12654 | 0.191285 | 0.169454 | 0.25849 |

Home with non-household members; urban

|  | 0 | 5 | 10 | 15 | 20 | 25 | 30 | 35 | 40 | 45 | 50 | 55 | 60 | 65 |
| --- | --- | --- | --- | --- | --- | --- | --- | --- | --- | --- | --- | --- | --- | --- |
| 0 | 0.997389 | 0.597731 | 0.340267 | 0.113978 | 0.332302 | 0.256581 | 0.175235 | 0.207841 | 0.092676 | 0.16285 | 0.180259 | 0.087422 | 0.105441 | 0.111091 |
| 5 | 0.644309 | 1.261692 | 0.638507 | 0.121226 | 0.170511 | 0.111063 | 0.135337 | 0.233061 | 0.115293 | 0.233298 | 0.107775 | 0.365441 | 0.375005 | 0.12856 |
| 10 | 0.366783 | 0.638507 | 1.913184 | 0.195024 | 0.229178 | 0.200625 | 0.169047 | 0.25287 | 0.106571 | 0.216362 | 0.299619 | 0.249489 | 0.257168 | 0.115609 |
| 15 | 0.135442 | 0.13364 | 0.214996 | 0.455727 | 0.407997 | 0.337893 | 0.412295 | 0.391317 | 0.1891 | 0.104648 | 0.350828 | 0.391177 | 0.22303 | 0.1931 |
| 20 | 0.500612 | 0.238305 | 0.320297 | 0.517243 | 0.481843 | 0.88965 | 0.562306 | 0.505246 | 0.654674 | 0.34314 | 0.776178 | 1.009588 | 0.380221 | 0.160709 |
| 25 | 0.328225 | 0.131804 | 0.238091 | 0.363743 | 0.755435 | 0.735632 | 0.698215 | 0.927676 | 0.5161 | 0.186684 | 0.572501 | 0.449279 | 0.312421 | 0.231418 |
| 30 | 0.221889 | 0.15898 | 0.198579 | 0.439332 | 0.472628 | 0.691127 | 0.474443 | 0.914666 | 0.63139 | 0.339549 | 0.394972 | 0.72882 | 0.480866 | 0.235129 |
| 35 | 0.147109 | 0.153034 | 0.166041 | 0.233081 | 0.237379 | 0.513284 | 0.511275 | 0.533987 | 0.207674 | 0.275687 | 0.293823 | 0.154338 | 0.164952 | 0.148149 |
| 40 | 0.081242 | 0.093762 | 0.086669 | 0.1395 | 0.380951 | 0.353672 | 0.437115 | 0.25721 | 0.348844 | 0.295238 | 0.175041 | 0.362967 | 0.30014 | 0.194847 |
| 45 | 0.117379 | 0.156001 | 0.144676 | 0.063475 | 0.164175 | 0.105188 | 0.193282 | 0.280745 | 0.242752 | 0.296012 | 0.283798 | 0.376396 | 0.187451 | 0.063028 |
| 50 | 0.119393 | 0.066223 | 0.184103 | 0.195544 | 0.341251 | 0.296422 | 0.206601 | 0.274953 | 0.132254 | 0.260787 | 0.217081 | 0.194353 | 0.104214 | 0.072007 |
| 55 | 0.045414 | 0.176117 | 0.120236 | 0.171007 | 0.348135 | 0.182449 | 0.299004 | 0.113276 | 0.215093 | 0.271277 | 0.152434 | 0.236168 | 0.108609 | 0.094 |
| 60 | 0.04245 | 0.140062 | 0.096051 | 0.075563 | 0.101611 | 0.098326 | 0.152891 | 0.093826 | 0.137843 | 0.104703 | 0.063346 | 0.084172 | 0.098943 | 0.060906 |
| 65 | 0.077908 | 0.083641 | 0.075215 | 0.113961 | 0.074812 | 0.126868 | 0.130225 | 0.146789 | 0.155877 | 0.061325 | 0.076243 | 0.126899 | 0.106093 | 0.239471 |

Home with non-household members; rural

|  | 0 | 5 | 10 | 15 | 20 | 25 | 30 | 35 | 40 | 45 | 50 | 55 | 60 | 65 |
| --- | --- | --- | --- | --- | --- | --- | --- | --- | --- | --- | --- | --- | --- | --- |
| 0 | 0.561318 | 0.776718 | 0.461975 | 0.252669 | 0.320731 | 0.266473 | 0.262773 | 0.470897 | 0.176269 | 0.488362 | 0.359042 | 0.435461 | 0.982824 | 0.298415 |
| 5 | 0.879143 | 1.544468 | 0.491541 | 0.321155 | 0.434401 | 0.413546 | 0.402378 | 0.723521 | 0.41208 | 0.53678 | 0.965808 | 0.795209 | 1.074403 | 0.530595 |
| 10 | 0.558432 | 0.524947 | 1.016523 | 0.542507 | 0.448091 | 0.426085 | 0.757859 | 0.938868 | 0.380012 | 0.766522 | 0.924331 | 0.646444 | 0.902156 | 0.668915 |
| 15 | 0.302648 | 0.339863 | 0.537575 | 0.596528 | 0.693112 | 0.885249 | 0.684588 | 1.103388 | 0.374539 | 0.525255 | 0.916712 | 0.45735 | 1.186895 | 0.43344 |
| 20 | 0.384172 | 0.459706 | 0.444017 | 0.693112 | 0.625259 | 0.758536 | 0.868236 | 1.139641 | 0.850728 | 0.900835 | 0.557049 | 0.508038 | 1.60986 | 0.503167 |
| 25 | 0.245976 | 0.337261 | 0.325374 | 0.682211 | 0.58456 | 0.543921 | 0.693155 | 0.608243 | 0.452656 | 0.985836 | 0.622398 | 0.984559 | 1.020288 | 0.441302 |
| 30 | 0.225234 | 0.304714 | 0.53739 | 0.489888 | 0.621306 | 0.643644 | 0.628934 | 1.307384 | 0.797576 | 0.590877 | 0.75821 | 0.681793 | 0.848421 | 0.438349 |
| 35 | 0.24321 | 0.330151 | 0.401153 | 0.475773 | 0.491405 | 0.340326 | 0.787783 | 0.757284 | 0.552791 | 0.524099 | 0.296257 | 0.641035 | 0.543007 | 0.307452 |
| 40 | 0.101694 | 0.210041 | 0.181369 | 0.180397 | 0.409755 | 0.28291 | 0.536831 | 0.617479 | 0.466572 | 0.460688 | 0.383801 | 0.329275 | 0.627568 | 0.19024 |
| 45 | 0.276381 | 0.26839 | 0.358871 | 0.248171 | 0.425623 | 0.604411 | 0.39013 | 0.574278 | 0.451912 | 0.59563 | 0.393055 | 0.641481 | 0.412072 | 0.255009 |
| 50 | 0.181493 | 0.431331 | 0.386538 | 0.386869 | 0.235085 | 0.340837 | 0.447149 | 0.289953 | 0.336282 | 0.351078 | 0.498996 | 0.348074 | 0.344618 | 0.172809 |
| 55 | 0.157914 | 0.254775 | 0.193933 | 0.138464 | 0.15381 | 0.38679 | 0.288451 | 0.450088 | 0.206973 | 0.411046 | 0.249705 | 0.185576 | 0.081281 | 0.095909 |
| 60 | 0.302408 | 0.292071 | 0.22964 | 0.304891 | 0.413543 | 0.340096 | 0.304562 | 0.323494 | 0.334703 | 0.22404 | 0.209768 | 0.068966 | 0.091563 | 0.183466 |
| 65 | 0.190198 | 0.298781 | 0.352701 | 0.230638 | 0.26774 | 0.304708 | 0.325952 | 0.379409 | 0.21017 | 0.287195 | 0.21789 | 0.168568 | 0.380037 | 0.208435 |

Work; urban

|  | 0 | 5 | 10 | 15 | 20 | 25 | 30 | 35 | 40 | 45 | 50 | 55 | 60 | 65 |
| --- | --- | --- | --- | --- | --- | --- | --- | --- | --- | --- | --- | --- | --- | --- |
| 0 | 0 | 0 | 0 | 0.009781 | 0 | 0.006401 | 0.006038 | 0.003019 | 0 | 0 | 0 | 0 | 0 | 0 |
| 5 | 0 | 0.015312 | 0 | 0 | 0.014364 | 0 | 0 | 0.003828 | 0 | 0 | 0 | 0 | 0 | 0 |
| 10 | 0 | 0 | 0 | 0.004677 | 0.04096 | 0.007716 | 0.003286 | 0.012855 | 0.103482 | 0.003286 | 0.003286 | 0 | 0 | 0 |
| 15 | 0.011623 | 0 | 0.005156 | 0.174609 | 0.129332 | 0.133826 | 0.028961 | 0.036379 | 0.008035 | 0 | 0 | 0.070617 | 0 | 0.00707 |
| 20 | 0 | 0.020075 | 0.057245 | 0.163963 | 0.278238 | 0.318345 | 0.166396 | 0.206934 | 0.109686 | 0.051356 | 0.068266 | 0.18526 | 0.018629 | 0.008963 |
| 25 | 0.008188 | 0 | 0.009157 | 0.144064 | 0.270319 | 0.517034 | 0.326581 | 0.140363 | 0.086739 | 0.215227 | 0.096107 | 0.047166 | 0.063576 | 0.022831 |
| 30 | 0.007645 | 0 | 0.00386 | 0.030861 | 0.139859 | 0.323266 | 0.171597 | 0.213339 | 0.257996 | 0.375143 | 0.351024 | 0.037624 | 0.062931 | 0.014286 |
| 35 | 0.002137 | 0.002514 | 0.008441 | 0.021669 | 0.097223 | 0.077663 | 0.119251 | 0.13506 | 0.194954 | 0.194223 | 0.162058 | 0.037913 | 0.052059 | 0.021074 |
| 40 | 0 | 0 | 0.084157 | 0.005928 | 0.063826 | 0.05944 | 0.178612 | 0.241455 | 0.262974 | 0.307469 | 0.059585 | 0.116752 | 0.087135 | 0.031292 |
| 45 | 0 | 0 | 0.002197 | 0 | 0.024571 | 0.12127 | 0.213543 | 0.197786 | 0.252808 | 0.111774 | 0.08625 | 0.060224 | 0.03654 | 0 |
| 50 | 0 | 0 | 0.002019 | 0 | 0.030013 | 0.049761 | 0.183613 | 0.15165 | 0.04502 | 0.079257 | 0.210094 | 0.04502 | 0.046472 | 0.015762 |
| 55 | 0 | 0 | 0 | 0.030871 | 0.063883 | 0.019154 | 0.015435 | 0.027826 | 0.069187 | 0.043405 | 0.03531 | 0 | 0.038727 | 0.003091 |
| 60 | 0 | 0 | 0 | 0 | 0.004978 | 0.020009 | 0.020009 | 0.029612 | 0.040018 | 0.02041 | 0.028248 | 0.030013 | 0 | 0 |
| 65 | 0 | 0 | 0 | 0.004172 | 0.004172 | 0.012517 | 0.007912 | 0.02088 | 0.025033 | 0 | 0.016689 | 0.004172 | 0 | 0.008344 |

Work; rural

|  | 0 | 5 | 10 | 15 | 20 | 25 | 30 | 35 | 40 | 45 | 50 | 55 | 60 | 65 |
| --- | --- | --- | --- | --- | --- | --- | --- | --- | --- | --- | --- | --- | --- | --- |
| 0 | 0 | 0.003154 | 0 | 0.003438 | 0.003438 | 0 | 0.003438 | 0 | 0.021282 | 0 | 0 | 0 | 0 | 0 |
| 5 | 0.00357 | 0.014282 | 0.005519 | 0.002512 | 0.00357 | 0 | 0.00357 | 0 | 0.03123 | 0 | 0 | 0.007141 | 0 | 0 |
| 10 | 0 | 0.005894 | 0.064835 | 0.025204 | 0.002947 | 0 | 0.005894 | 0.040535 | 0.002947 | 0 | 0.002947 | 0 | 0.002947 | 0 |
| 15 | 0.004118 | 0.002659 | 0.024975 | 0.062829 | 0.011255 | 0.044089 | 0.005937 | 0.068652 | 0.025492 | 0 | 0 | 0.068632 | 0.016841 | 0 |
| 20 | 0.004118 | 0.003778 | 0.00292 | 0.011255 | 0 | 0.050073 | 0 | 0.154467 | 0.025492 | 0.030903 | 0 | 0.068632 | 0.016841 | 0 |
| 25 | 0 | 0 | 0 | 0.033977 | 0.038589 | 0.015435 | 0.023153 | 0.134474 | 0.078361 | 0.023815 | 0.035904 | 0.170985 | 0.028414 | 0.012222 |
| 30 | 0.002947 | 0.002704 | 0.004179 | 0.004248 | 0 | 0.021499 | 0.015659 | 0.098254 | 0.00783 | 0.088457 | 0 | 0.378089 | 0.061662 | 0.028373 |
| 35 | 0 | 0 | 0.017319 | 0.029602 | 0.066605 | 0.075242 | 0.059204 | 0.074006 | 0.04358 | 0.028126 | 0.014801 | 0.103079 | 0.076037 | 0.006839 |
| 40 | 0.012278 | 0.015918 | 0.001407 | 0.012278 | 0.012278 | 0.048975 | 0.00527 | 0.04868 | 0 | 0.103428 | 0.012278 | 0.07961 | 0.153336 | 0.011458 |
| 45 | 0 | 0 | 0 | 0 | 0.014601 | 0.014601 | 0.058404 | 0.030819 | 0.101458 | 0 | 0 | 0.014601 | 0.089809 | 0 |
| 50 | 0 | 0 | 0.001232 | 0 | 0 | 0.019662 | 0 | 0.014486 | 0.010758 | 0 | 0.061742 | 0 | 0.134352 | 0.015435 |
| 55 | 0 | 0.002288 | 0 | 0.020778 | 0.020778 | 0.067173 | 0.159961 | 0.072375 | 0.05004 | 0.009356 | 0 | 0 | 0.138744 | 0 |
| 60 | 0 | 0 | 0.00075 | 0.004326 | 0.004326 | 0.009471 | 0.022135 | 0.045299 | 0.081779 | 0.048828 | 0.081779 | 0.117723 | 0.04089 | 0.065409 |
| 65 | 0 | 0 | 0 | 0 | 0 | 0.008439 | 0.021098 | 0.008439 | 0.012659 | 0 | 0.019462 | 0 | 0.135489 | 0 |

School; urban

|  | 0 | 5 | 10 | 15 | 20 | 25 | 30 | 35 | 40 | 45 | 50 | 55 | 60 | 65 |
| --- | --- | --- | --- | --- | --- | --- | --- | --- | --- | --- | --- | --- | --- | --- |
| 0 | 0 | 0.003551 | 0 | 0 | 0 | 0 | 0 | 0 | 0 | 0 | 0 | 0 | 0.049699 | 0 |
| 5 | 0.003828 | 1.622789 | 0.313495 | 0.030319 | 0.043753 | 0.007656 | 0.038307 | 0.011484 | 0.016204 | 0.007656 | 0.003828 | 0 | 0 | 0 |
| 10 | 0 | 0.313495 | 2.412666 | 0.198074 | 0.029575 | 0.033626 | 0.033626 | 0.181608 | 0.034391 | 0.01796 | 0.006572 | 0.013909 | 0 | 0 |
| 15 | 0 | 0.033424 | 0.218358 | 1.423838 | 0.359025 | 0.040474 | 0.012891 | 0.038796 | 0.041375 | 0.01667 | 0 | 0.005757 | 0.029529 | 0 |
| 20 | 0 | 0.061149 | 0.041333 | 0.455159 | 0.425929 | 0.070254 | 0.017939 | 0.008801 | 0 | 0.008801 | 0.013865 | 0.013865 | 0 | 0 |
| 25 | 0 | 0.009086 | 0.039905 | 0.04357 | 0.059655 | 0.017305 | 0.034792 | 0 | 0 | 0 | 0 | 0 | 0.031788 | 0 |
| 30 | 0 | 0.045 | 0.0395 | 0.013736 | 0.015078 | 0.034438 | 0.094542 | 0.015101 | 0.021831 | 0 | 0 | 0 | 0.062931 | 0 |
| 35 | 0 | 0.007541 | 0.119249 | 0.023108 | 0.004135 | 0 | 0.008441 | 0 | 0.010144 | 0 | 0.008441 | 0.008441 | 0 | 0 |
| 40 | 0 | 0.013178 | 0.027968 | 0.030523 | 0 | 0 | 0.015114 | 0.012564 | 0 | 0.012564 | 0 | 0 | 0.021784 | 0 |
| 45 | 0 | 0.00512 | 0.01201 | 0.010111 | 0.004211 | 0 | 0 | 0 | 0.01033 | 0 | 0 | 0 | 0 | 0 |
| 50 | 0 | 0.002352 | 0.004038 | 0 | 0.006096 | 0 | 0 | 0.007899 | 0 | 0 | 0 | 0 | 0 | 0 |
| 55 | 0 | 0 | 0.006703 | 0.002517 | 0.004781 | 0 | 0 | 0.006195 | 0 | 0 | 0 | 0 | 0.012909 | 0 |
| 60 | 0.020009 | 0 | 0 | 0.010004 | 0 | 0.010004 | 0.020009 | 0 | 0.010004 | 0 | 0 | 0.010004 | 0 | 0 |
| 65 | 0 | 0 | 0 | 0 | 0 | 0 | 0 | 0 | 0 | 0 | 0 | 0 | 0 | 0 |

School; rural

|  | 0 | 5 | 10 | 15 | 20 | 25 | 30 | 35 | 40 | 45 | 50 | 55 | 60 | 65 |
| --- | --- | --- | --- | --- | --- | --- | --- | --- | --- | --- | --- | --- | --- | --- |
| 0 | 0.006876 | 0.107118 | 0.023067 | 0.004439 | 0 | 0 | 0 | 0.132397 | 0 | 0 | 0 | 0 | 0 | 0.013241 |
| 5 | 0.121244 | 2.353729 | 0.24837 | 0.024331 | 0.014282 | 0.010711 | 0.014282 | 0.102514 | 0.010711 | 0.00357 | 0.00357 | 0 | 0 | 0.015465 |
| 10 | 0.027883 | 0.265249 | 2.471043 | 0.295585 | 0.005894 | 0.005894 | 0.011788 | 0.005894 | 0.008841 | 0.014735 | 0 | 0.005894 | 0.002947 | 0 |
| 15 | 0.005318 | 0.025749 | 0.292898 | 1.119671 | 0.108077 | 0.002659 | 0.019849 | 0.076643 | 0 | 0 | 0 | 0 | 0.002659 | 0 |
| 20 | 0 | 0.015114 | 0.00584 | 0.108077 | 0.06993 | 0.003496 | 0 | 0.003496 | 0.050984 | 0 | 0 | 0 | 0 | 0 |
| 25 | 0 | 0.008735 | 0.004501 | 0.002049 | 0.002695 | 0 | 0 | 0 | 0 | 0 | 0 | 0 | 0 | 0.006111 |
| 30 | 0 | 0.010815 | 0.008359 | 0.014204 | 0 | 0 | 0 | 0 | 0.018242 | 0 | 0 | 0 | 0 | 0.005675 |
| 35 | 0.068381 | 0.046778 | 0.002518 | 0.033048 | 0.001508 | 0 | 0 | 0 | 0.010992 | 0 | 0 | 0 | 0 | 0 |
| 40 | 0 | 0.00546 | 0.00422 | 0 | 0.024556 | 0 | 0.012278 | 0.012278 | 0.098226 | 0 | 0 | 0 | 0 | 0 |
| 45 | 0 | 0.001785 | 0.006899 | 0 | 0 | 0 | 0 | 0 | 0 | 0 | 0 | 0 | 0 | 0 |
| 50 | 0 | 0.001595 | 0 | 0 | 0 | 0 | 0 | 0 | 0 | 0 | 0 | 0 | 0 | 0 |
| 55 | 0 | 0 | 0.001768 | 0 | 0 | 0 | 0 | 0 | 0 | 0 | 0 | 0 | 0 | 0 |
| 60 | 0 | 0 | 0.00075 | 0.000683 | 0 | 0 | 0 | 0 | 0 | 0 | 0 | 0 | 0 | 0.002037 |
| 65 | 0.008439 | 0.008709 | 0 | 0 | 0 | 0.00422 | 0.00422 | 0 | 0 | 0 | 0 | 0 | 0.00422 | 0 |

Other (including transport and leisure); urban

|  | 0 | 5 | 10 | 15 | 20 | 25 | 30 | 35 | 40 | 45 | 50 | 55 | 60 | 65 |
| --- | --- | --- | --- | --- | --- | --- | --- | --- | --- | --- | --- | --- | --- | --- |
| 0 | 0.140275 | 0.096588 | 0.111705 | 0.049753 | 0.061531 | 0.054317 | 0.033909 | 0.045445 | 0.059556 | 0.018816 | 0.030466 | 0.052251 | 0.012076 | 0.021658 |
| 5 | 0.104115 | 0.377138 | 0.224728 | 0.070435 | 0.009755 | 0.066789 | 0.021377 | 0.059304 | 0.065623 | 0.008547 | 0.011539 | 0.100214 | 0.040912 | 0.010241 |
| 10 | 0.12041 | 0.224728 | 0.400448 | 0.094013 | 0.096522 | 0.042719 | 0.072286 | 0.146213 | 0.068574 | 0.009858 | 0.021246 | 0.103181 | 0.056637 | 0.030021 |
| 15 | 0.059122 | 0.077648 | 0.10364 | 0.470595 | 0.389566 | 0.259326 | 0.164882 | 0.281298 | 0.243711 | 0.143228 | 0.135276 | 0.168529 | 0.126152 | 0.024753 |
| 20 | 0.092697 | 0.013634 | 0.134898 | 0.493877 | 0.397524 | 0.597497 | 0.213229 | 0.513278 | 0.35087 | 0.266945 | 0.515511 | 0.068845 | 0.420333 | 0.070692 |
| 25 | 0.069484 | 0.079262 | 0.050696 | 0.279166 | 0.507357 | 0.993783 | 0.671628 | 0.598757 | 0.365615 | 0.342264 | 0.239475 | 0.327119 | 0.489421 | 0.068494 |
| 30 | 0.042937 | 0.025111 | 0.084914 | 0.175695 | 0.179223 | 0.66481 | 0.451239 | 0.74659 | 0.526495 | 0.392387 | 0.348311 | 0.604483 | 0.642394 | 0.058705 |
| 35 | 0.032166 | 0.038941 | 0.096007 | 0.16755 | 0.241153 | 0.331293 | 0.417325 | 0.416968 | 0.429289 | 0.2752 | 0.251935 | 0.301492 | 0.240106 | 0.078838 |
| 40 | 0.052208 | 0.053368 | 0.055768 | 0.179787 | 0.20417 | 0.250548 | 0.364496 | 0.531685 | 0.538165 | 0.221286 | 0.299879 | 0.132383 | 0.367424 | 0.054286 |
| 45 | 0.013562 | 0.005715 | 0.006592 | 0.086876 | 0.12772 | 0.192849 | 0.223359 | 0.280249 | 0.181947 | 0.493027 | 0.081019 | 0.142729 | 0.354204 | 0.017152 |
| 50 | 0.020179 | 0.00709 | 0.013055 | 0.0754 | 0.226647 | 0.123992 | 0.182193 | 0.235756 | 0.226576 | 0.07445 | 0.097027 | 0.069374 | 0.403465 | 0.052829 |
| 55 | 0.027144 | 0.048296 | 0.049726 | 0.073674 | 0.02374 | 0.132841 | 0.247994 | 0.221279 | 0.07845 | 0.102868 | 0.054411 | 0.121783 | 0.203783 | 0.03424 |
| 60 | 0.004862 | 0.015281 | 0.021154 | 0.04274 | 0.112331 | 0.154031 | 0.204249 | 0.136574 | 0.168744 | 0.197844 | 0.245244 | 0.157932 | 0.21406 | 0.176411 |
| 65 | 0.015189 | 0.006663 | 0.019532 | 0.014608 | 0.032908 | 0.03755 | 0.032513 | 0.078115 | 0.043429 | 0.016689 | 0.055937 | 0.046224 | 0.307294 | 0.202208 |

Other (including transport and leisure); rural

|  | 0 | 5 | 10 | 15 | 20 | 25 | 30 | 35 | 40 | 45 | 50 | 55 | 60 | 65 |
| --- | --- | --- | --- | --- | --- | --- | --- | --- | --- | --- | --- | --- | --- | --- |
| 0 | 0.403634 | 0.626744 | 0.476315 | 0.377037 | 0.256131 | 0.292798 | 0.212337 | 0.19853 | 0.222837 | 0.192489 | 0.136475 | 0.253261 | 0.198089 | 0.087378 |
| 5 | 0.709392 | 1.461915 | 0.685954 | 0.489107 | 0.486209 | 0.393004 | 0.423865 | 0.562845 | 0.191606 | 0.317015 | 0.518881 | 0.403407 | 0.17193 | 0.162306 |
| 10 | 0.575766 | 0.732572 | 1.449592 | 0.784251 | 0.57275 | 0.373871 | 0.786074 | 0.786198 | 0.564746 | 0.914611 | 0.537673 | 0.43052 | 0.276986 | 0.207401 |
| 15 | 0.451616 | 0.517598 | 0.777121 | 1.148121 | 1.048729 | 1.023954 | 0.898439 | 0.882295 | 0.77289 | 0.991387 | 1.291687 | 0.926311 | 0.578661 | 0.234033 |
| 20 | 0.306795 | 0.514532 | 0.567543 | 1.048729 | 1.783353 | 1.450915 | 1.381455 | 1.295529 | 1.086923 | 1.648713 | 1.256853 | 1.080117 | 0.761773 | 0.377035 |
| 25 | 0.270275 | 0.320508 | 0.285502 | 0.789102 | 1.118137 | 1.538213 | 1.272667 | 1.400703 | 1.012897 | 1.460406 | 1.005748 | 1.558363 | 0.946337 | 0.349996 |
| 30 | 0.182003 | 0.320985 | 0.557397 | 0.642919 | 0.988564 | 1.181761 | 1.325227 | 1.284981 | 0.987589 | 1.647039 | 1.338873 | 1.716677 | 1.12853 | 0.467266 |
| 35 | 0.102538 | 0.256832 | 0.335921 | 0.380439 | 0.558623 | 0.783727 | 0.774284 | 0.686724 | 0.583876 | 0.930602 | 0.97143 | 0.895828 | 0.411386 | 0.228791 |
| 40 | 0.12856 | 0.097663 | 0.269538 | 0.372264 | 0.523518 | 0.63306 | 0.664724 | 0.652202 | 0.545957 | 0.601506 | 0.855399 | 0.674198 | 0.58788 | 0.249922 |
| 45 | 0.108936 | 0.158508 | 0.428204 | 0.468408 | 0.778978 | 0.895367 | 1.087468 | 1.0197 | 0.590048 | 0.386427 | 0.917162 | 0.596128 | 0.65769 | 0.289354 |
| 50 | 0.068987 | 0.231733 | 0.224845 | 0.545115 | 0.530415 | 0.550766 | 0.789592 | 0.95076 | 0.749492 | 0.819213 | 0.80983 | 0.598572 | 0.484593 | 0.538495 |
| 55 | 0.091842 | 0.129247 | 0.129156 | 0.280443 | 0.327008 | 0.612213 | 0.726286 | 0.628984 | 0.423781 | 0.381985 | 0.42941 | 0.764514 | 0.31475 | 0.256999 |
| 60 | 0.06095 | 0.046738 | 0.070506 | 0.148647 | 0.195685 | 0.315446 | 0.405114 | 0.245081 | 0.313536 | 0.35758 | 0.29497 | 0.267062 | 0.44323 | 0.297009 |
| 65 | 0.055691 | 0.091396 | 0.109357 | 0.124531 | 0.200624 | 0.241664 | 0.347454 | 0.282337 | 0.276104 | 0.325875 | 0.678973 | 0.451695 | 0.615231 | 0.477909 |

All; urban; physical or ≥15 mins

|  | 0 | 5 | 10 | 15 | 20 | 25 | 30 | 35 | 40 | 45 | 50 | 55 | 60 | 65 |
| --- | --- | --- | --- | --- | --- | --- | --- | --- | --- | --- | --- | --- | --- | --- |
| 0 | 1.439139 | 1.063077 | 0.776515 | 0.369297 | 0.622848 | 0.820241 | 0.757523 | 0.647992 | 0.518748 | 0.373003 | 0.458978 | 0.415807 | 0.506165 | 0.319344 |
| 5 | 1.145918 | 3.588527 | 1.516636 | 0.409906 | 0.398131 | 0.531876 | 0.805064 | 0.734849 | 0.603625 | 0.623604 | 0.346813 | 0.714369 | 0.611196 | 0.356797 |
| 10 | 0.837025 | 1.516636 | 4.908335 | 0.803498 | 0.61369 | 0.444867 | 0.491482 | 1.103204 | 0.815282 | 0.916826 | 0.685209 | 0.487787 | 0.733078 | 0.399655 |
| 15 | 0.43884 | 0.451883 | 0.885781 | 2.590249 | 1.504449 | 1.102447 | 0.760273 | 1.297551 | 0.851487 | 0.963243 | 1.032758 | 1.23254 | 0.981451 | 0.666383 |
| 20 | 0.938319 | 0.556424 | 0.857687 | 1.907285 | 1.870771 | 2.030726 | 1.147653 | 1.417231 | 1.132975 | 1.063291 | 1.812601 | 1.21712 | 1.668822 | 0.56912 |
| 25 | 1.049271 | 0.631201 | 0.527944 | 1.186789 | 1.724365 | 2.327701 | 1.843125 | 1.465425 | 0.836853 | 0.806786 | 1.361971 | 1.117619 | 1.410754 | 0.477496 |
| 30 | 0.959204 | 0.945707 | 0.577343 | 0.810129 | 0.964622 | 1.824414 | 1.256287 | 1.580264 | 1.028665 | 1.023827 | 1.038164 | 1.516664 | 1.342543 | 0.604553 |
| 35 | 0.458646 | 0.482522 | 0.724394 | 0.772862 | 0.665855 | 0.810821 | 0.883328 | 1.07792 | 0.839647 | 0.658802 | 0.668966 | 0.528863 | 0.518588 | 0.327716 |
| 40 | 0.454747 | 0.490898 | 0.663029 | 0.628146 | 0.659272 | 0.573477 | 0.712151 | 1.039925 | 1.132258 | 0.878324 | 0.477121 | 0.317295 | 0.65005 | 0.546296 |
| 45 | 0.268854 | 0.416988 | 0.613058 | 0.584264 | 0.50873 | 0.454585 | 0.582794 | 0.670889 | 0.72218 | 0.870177 | 0.550743 | 0.286412 | 0.427974 | 0.295493 |
| 50 | 0.303999 | 0.213102 | 0.421032 | 0.575637 | 0.796919 | 0.705183 | 0.543039 | 0.626004 | 0.360493 | 0.506088 | 0.656782 | 0.250361 | 0.515248 | 0.330708 |
| 55 | 0.216005 | 0.344275 | 0.235079 | 0.538818 | 0.419698 | 0.453857 | 0.622223 | 0.388157 | 0.188028 | 0.206423 | 0.196362 | 0.310795 | 0.540697 | 0.217994 |
| 60 | 0.203782 | 0.228279 | 0.273801 | 0.332515 | 0.44598 | 0.443996 | 0.426861 | 0.294977 | 0.298543 | 0.23905 | 0.313191 | 0.41904 | 0.448269 | 0.282866 |
| 65 | 0.223955 | 0.232132 | 0.260016 | 0.393275 | 0.264934 | 0.261774 | 0.334828 | 0.324707 | 0.437036 | 0.287506 | 0.35016 | 0.294289 | 0.492731 | 0.547639 |

All; rural; physical or ≥15 mins

|  | 0 | 5 | 10 | 15 | 20 | 25 | 30 | 35 | 40 | 45 | 50 | 55 | 60 | 65 |
| --- | --- | --- | --- | --- | --- | --- | --- | --- | --- | --- | --- | --- | --- | --- |
| 0 | 1.044267 | 1.832654 | 1.140579 | 0.706933 | 0.962215 | 1.218488 | 1.131166 | 1.333514 | 0.853659 | 1.116982 | 0.793437 | 0.963798 | 1.492005 | 0.612277 |
| 5 | 2.074323 | 5.399633 | 1.824153 | 0.995292 | 0.95219 | 1.324199 | 1.470547 | 1.962359 | 1.337122 | 1.098266 | 1.519009 | 1.237661 | 1.572147 | 0.872272 |
| 10 | 1.378723 | 1.948125 | 4.757031 | 1.80571 | 1.03324 | 0.845152 | 1.506209 | 2.525873 | 1.606682 | 1.983852 | 1.596562 | 1.088753 | 1.276656 | 0.976008 |
| 15 | 0.846766 | 1.05327 | 1.789294 | 2.818284 | 1.987496 | 1.931012 | 1.044216 | 2.331254 | 1.678061 | 2.287481 | 2.914678 | 1.611168 | 2.388895 | 0.765784 |
| 20 | 1.152543 | 1.007657 | 1.023846 | 1.987496 | 2.573435 | 2.214509 | 1.573695 | 1.910226 | 2.018255 | 2.523677 | 2.583475 | 2.316948 | 2.867901 | 0.882566 |
| 25 | 1.124758 | 1.079929 | 0.645389 | 1.48812 | 1.706595 | 1.799765 | 1.696464 | 1.428024 | 1.157334 | 1.847831 | 1.78842 | 1.814657 | 1.990193 | 0.613072 |
| 30 | 0.969571 | 1.113617 | 1.068038 | 0.747237 | 1.12613 | 1.575287 | 1.762606 | 1.895709 | 1.267583 | 1.510934 | 1.599042 | 1.677526 | 1.196674 | 0.681365 |
| 35 | 0.688739 | 0.895446 | 1.079237 | 1.00522 | 0.823676 | 0.799014 | 1.142287 | 1.576301 | 0.950621 | 1.152221 | 0.746486 | 0.572855 | 0.657161 | 0.533724 |
| 40 | 0.492496 | 0.681543 | 0.766825 | 0.808241 | 0.972096 | 0.723334 | 0.853181 | 1.061864 | 1.115288 | 0.923647 | 0.814387 | 0.610864 | 0.941763 | 0.352587 |
| 45 | 0.632138 | 0.549132 | 0.928802 | 1.080782 | 1.192379 | 1.132895 | 0.997604 | 1.262538 | 0.906052 | 1.074853 | 1.118395 | 0.481593 | 0.725536 | 0.420354 |
| 50 | 0.401078 | 0.678392 | 0.667653 | 1.230047 | 1.090273 | 0.979372 | 0.943025 | 0.730602 | 0.713557 | 0.998955 | 1.738894 | 0.802998 | 0.822425 | 0.689927 |
| 55 | 0.349509 | 0.396532 | 0.326625 | 0.487784 | 0.701461 | 0.7129 | 0.709722 | 0.402216 | 0.383971 | 0.308593 | 0.576063 | 0.933309 | 0.568379 | 0.327223 |
| 60 | 0.459079 | 0.42738 | 0.324967 | 0.613662 | 0.73671 | 0.663398 | 0.429576 | 0.3915 | 0.502274 | 0.394467 | 0.500607 | 0.482262 | 0.571065 | 0.515762 |
| 65 | 0.390243 | 0.491182 | 0.514622 | 0.407481 | 0.469622 | 0.423311 | 0.506656 | 0.658638 | 0.389524 | 0.473409 | 0.869909 | 0.575121 | 1.068364 | 0.736399 |

Home with household members; urban; physical or ≥15 mins

|  | 0 | 5 | 10 | 15 | 20 | 25 | 30 | 35 | 40 | 45 | 50 | 55 | 60 | 65 |
| --- | --- | --- | --- | --- | --- | --- | --- | --- | --- | --- | --- | --- | --- | --- |
| 0 | 0.301475 | 0.374264 | 0.336648 | 0.23975 | 0.261486 | 0.548458 | 0.564176 | 0.404466 | 0.406406 | 0.207134 | 0.280792 | 0.285894 | 0.33895 | 0.192544 |
| 5 | 0.403428 | 0.349876 | 0.384474 | 0.221549 | 0.191229 | 0.370287 | 0.640997 | 0.526228 | 0.525899 | 0.353899 | 0.246694 | 0.280743 | 0.355995 | 0.245141 |
| 10 | 0.362881 | 0.384474 | 0.426284 | 0.377108 | 0.317143 | 0.257746 | 0.28509 | 0.697723 | 0.661941 | 0.744708 | 0.383069 | 0.188551 | 0.483468 | 0.308933 |
| 15 | 0.284898 | 0.244237 | 0.415727 | 0.321402 | 0.42832 | 0.515229 | 0.298023 | 0.770434 | 0.578323 | 0.771036 | 0.717338 | 0.881506 | 0.720856 | 0.509976 |
| 20 | 0.393928 | 0.267259 | 0.443236 | 0.543008 | 0.536704 | 0.684052 | 0.449765 | 0.69649 | 0.540665 | 0.7381 | 0.904691 | 0.901453 | 1.132311 | 0.433604 |
| 25 | 0.7016 | 0.439437 | 0.305878 | 0.554646 | 0.580854 | 0.660491 | 0.539519 | 0.364121 | 0.225218 | 0.266653 | 0.770828 | 0.7426 | 0.948577 | 0.304441 |
| 30 | 0.714381 | 0.752978 | 0.334895 | 0.317566 | 0.378036 | 0.534042 | 0.419413 | 0.30788 | 0.338364 | 0.320316 | 0.443673 | 0.679912 | 0.693759 | 0.394569 |
| 35 | 0.286279 | 0.345535 | 0.458144 | 0.458894 | 0.327231 | 0.201468 | 0.172097 | 0.324579 | 0.31403 | 0.12788 | 0.16413 | 0.143067 | 0.253158 | 0.171106 |
| 40 | 0.356265 | 0.427688 | 0.538324 | 0.426632 | 0.31461 | 0.154337 | 0.234251 | 0.388934 | 0.526116 | 0.244029 | 0.157782 | 0.05066 | 0.183999 | 0.334885 |
| 45 | 0.149298 | 0.236643 | 0.497967 | 0.467679 | 0.353143 | 0.150246 | 0.182334 | 0.130226 | 0.200647 | 0.350617 | 0.163277 | 0.080091 | 0.11983 | 0.24204 |
| 50 | 0.18598 | 0.151583 | 0.23538 | 0.399829 | 0.397752 | 0.399109 | 0.232075 | 0.15359 | 0.119214 | 0.150039 | 0.349661 | 0.170654 | 0.101133 | 0.227408 |
| 55 | 0.148517 | 0.135298 | 0.090868 | 0.38536 | 0.310847 | 0.301565 | 0.278939 | 0.105004 | 0.030021 | 0.057724 | 0.133847 | 0.090912 | 0.309052 | 0.100137 |
| 60 | 0.136461 | 0.132962 | 0.180573 | 0.244226 | 0.302601 | 0.298538 | 0.22058 | 0.143998 | 0.084504 | 0.066932 | 0.061473 | 0.239515 | 0.174183 | 0.10399 |
| 65 | 0.135031 | 0.159488 | 0.200992 | 0.300969 | 0.20185 | 0.166901 | 0.21853 | 0.169536 | 0.267907 | 0.235497 | 0.240784 | 0.135183 | 0.181142 | 0.208036 |

Home with household members; rural; physical or ≥15 mins

|  | 0 | 5 | 10 | 15 | 20 | 25 | 30 | 35 | 40 | 45 | 50 | 55 | 60 | 65 |
| --- | --- | --- | --- | --- | --- | --- | --- | --- | --- | --- | --- | --- | --- | --- |
| 0 | 0.17191 | 0.47283 | 0.296396 | 0.23279 | 0.46444 | 0.748284 | 0.728956 | 0.582803 | 0.490536 | 0.534961 | 0.338771 | 0.332375 | 0.646708 | 0.241267 |
| 5 | 0.535181 | 0.429624 | 0.590016 | 0.363296 | 0.230179 | 0.648676 | 0.901649 | 0.966924 | 0.876132 | 0.510861 | 0.239265 | 0.389125 | 0.605779 | 0.33329 |
| 10 | 0.358281 | 0.630115 | 0.351866 | 0.568198 | 0.317242 | 0.222132 | 0.629473 | 1.363742 | 1.001586 | 0.854211 | 0.59676 | 0.464112 | 0.761924 | 0.399907 |
| 15 | 0.278836 | 0.384459 | 0.563032 | 0.476221 | 0.528492 | 0.377547 | 0.267188 | 1.241483 | 0.990555 | 1.344511 | 1.764893 | 1.383784 | 1.264817 | 0.307272 |
| 20 | 0.556308 | 0.243587 | 0.314358 | 0.528492 | 0.68337 | 0.493867 | 0.245576 | 0.29117 | 0.636457 | 0.696505 | 1.3298 | 1.140567 | 1.810889 | 0.298585 |
| 25 | 0.690725 | 0.529018 | 0.169628 | 0.290954 | 0.380595 | 0.529229 | 0.490187 | 0.225784 | 0.154046 | 0.334098 | 0.557374 | 0.919197 | 0.809688 | 0.211635 |
| 30 | 0.624819 | 0.682802 | 0.446354 | 0.191199 | 0.175733 | 0.455173 | 0.613275 | 0.342909 | 0.185199 | 0.094851 | 0.245073 | 0.481738 | 0.560084 | 0.299298 |
| 35 | 0.301008 | 0.441218 | 0.58269 | 0.535319 | 0.12555 | 0.126332 | 0.206625 | 0.497857 | 0.202741 | 0.079479 | 0.037973 | 0.117872 | 0.227753 | 0.229784 |
| 40 | 0.283002 | 0.446572 | 0.47803 | 0.477103 | 0.30655 | 0.096279 | 0.124653 | 0.226466 | 0.354039 | 0.181056 | 0.175287 | 0.012278 | 0.165867 | 0.101637 |
| 45 | 0.302753 | 0.25543 | 0.399925 | 0.63525 | 0.329083 | 0.204834 | 0.062626 | 0.087088 | 0.177607 | 0.537628 | 0.362962 | 0.047922 | 0.088129 | 0.09478 |
| 50 | 0.171247 | 0.106856 | 0.249554 | 0.744817 | 0.5612 | 0.305228 | 0.14453 | 0.037165 | 0.153585 | 0.324199 | 0.529867 | 0.145991 | 0.190771 | 0.100359 |
| 55 | 0.120531 | 0.124671 | 0.139233 | 0.418943 | 0.345309 | 0.361112 | 0.203812 | 0.082761 | 0.007718 | 0.030707 | 0.104733 | 0.488717 | 0.203902 | 0.108834 |
| 60 | 0.198987 | 0.164678 | 0.193945 | 0.324908 | 0.465183 | 0.269896 | 0.201056 | 0.135683 | 0.088463 | 0.047915 | 0.116122 | 0.173008 | 0.30053 | 0.081806 |
| 65 | 0.153775 | 0.187678 | 0.21086 | 0.163503 | 0.15888 | 0.146129 | 0.222555 | 0.283563 | 0.112284 | 0.106742 | 0.12654 | 0.191285 | 0.169454 | 0.25849 |

Home with non-household members; urban; physical or ≥15 mins

|  | 0 | 5 | 10 | 15 | 20 | 25 | 30 | 35 | 40 | 45 | 50 | 55 | 60 | 65 |
| --- | --- | --- | --- | --- | --- | --- | --- | --- | --- | --- | --- | --- | --- | --- |
| 0 | 0.997389 | 0.591693 | 0.331181 | 0.077624 | 0.299831 | 0.224206 | 0.1534 | 0.201803 | 0.073157 | 0.147053 | 0.161443 | 0.087422 | 0.105441 | 0.111091 |
| 5 | 0.637801 | 1.231068 | 0.607625 | 0.106281 | 0.156187 | 0.094859 | 0.118104 | 0.151394 | 0.042246 | 0.225642 | 0.096291 | 0.365441 | 0.214289 | 0.101416 |
| 10 | 0.356988 | 0.607625 | 1.819644 | 0.16302 | 0.181281 | 0.130115 | 0.139032 | 0.164825 | 0.087836 | 0.141013 | 0.277608 | 0.182146 | 0.20031 | 0.083385 |
| 15 | 0.092241 | 0.117165 | 0.179715 | 0.380531 | 0.324541 | 0.242894 | 0.30931 | 0.261871 | 0.07161 | 0.071357 | 0.209234 | 0.285252 | 0.190923 | 0.141302 |
| 20 | 0.451695 | 0.218285 | 0.253356 | 0.411441 | 0.403511 | 0.686657 | 0.415277 | 0.318462 | 0.335327 | 0.172121 | 0.515065 | 0.113608 | 0.250226 | 0.084836 |
| 25 | 0.28681 | 0.112574 | 0.154413 | 0.261477 | 0.583066 | 0.622498 | 0.596399 | 0.606885 | 0.373326 | 0.135309 | 0.406525 | 0.203525 | 0.213565 | 0.135003 |
| 30 | 0.194241 | 0.138736 | 0.16332 | 0.329593 | 0.349047 | 0.590345 | 0.349745 | 0.636096 | 0.283783 | 0.296878 | 0.180611 | 0.367597 | 0.325277 | 0.144526 |
| 35 | 0.142835 | 0.099409 | 0.108229 | 0.155979 | 0.149623 | 0.33579 | 0.355562 | 0.415809 | 0.105184 | 0.151866 | 0.19593 | 0.124866 | 0.095435 | 0.113481 |
| 40 | 0.064131 | 0.034356 | 0.071433 | 0.052827 | 0.195125 | 0.255832 | 0.196465 | 0.130273 | 0.242485 | 0.254622 | 0.120668 | 0.111529 | 0.147806 | 0.151911 |
| 45 | 0.105993 | 0.150881 | 0.094292 | 0.043282 | 0.082351 | 0.07624 | 0.168992 | 0.154652 | 0.209356 | 0.072464 | 0.257453 | 0.108839 | 0.075814 | 0.036301 |
| 50 | 0.10693 | 0.059167 | 0.170578 | 0.116623 | 0.226451 | 0.210485 | 0.094473 | 0.183347 | 0.091172 | 0.236579 | 0.12704 | 0 | 0.087755 | 0.038649 |
| 55 | 0.045414 | 0.176117 | 0.087781 | 0.124701 | 0.039175 | 0.08265 | 0.150809 | 0.091645 | 0.066092 | 0.078443 | 0 | 0.190712 | 0.063982 | 0.087819 |
| 60 | 0.04245 | 0.080036 | 0.074815 | 0.064685 | 0.066871 | 0.067214 | 0.103422 | 0.054284 | 0.067882 | 0.042347 | 0.053342 | 0.049586 | 0.06948 | 0.030217 |
| 65 | 0.077908 | 0.065981 | 0.05425 | 0.083392 | 0.039492 | 0.074012 | 0.080045 | 0.11244 | 0.121528 | 0.03532 | 0.040923 | 0.118555 | 0.052636 | 0.197749 |

Home with non-household members; rural; physical or ≥15 mins

|  | 0 | 5 | 10 | 15 | 20 | 25 | 30 | 35 | 40 | 45 | 50 | 55 | 60 | 65 |
| --- | --- | --- | --- | --- | --- | --- | --- | --- | --- | --- | --- | --- | --- | --- |
| 0 | 0.518459 | 0.71168 | 0.415841 | 0.230038 | 0.304059 | 0.259597 | 0.241344 | 0.456343 | 0.169393 | 0.459124 | 0.32163 | 0.435461 | 0.75029 | 0.270392 |
| 5 | 0.805528 | 1.350494 | 0.464273 | 0.281786 | 0.371109 | 0.394793 | 0.301734 | 0.621007 | 0.353191 | 0.53678 | 0.851411 | 0.578408 | 0.817494 | 0.409389 |
| 10 | 0.502665 | 0.495825 | 0.938528 | 0.468591 | 0.355344 | 0.375731 | 0.545502 | 0.738417 | 0.291047 | 0.763575 | 0.768095 | 0.344432 | 0.381307 | 0.434153 |
| 15 | 0.27554 | 0.298201 | 0.464331 | 0.507732 | 0.569635 | 0.750736 | 0.387934 | 0.868254 | 0.264285 | 0.285475 | 0.410458 | 0.008286 | 0.941953 | 0.336122 |
| 20 | 0.364203 | 0.392727 | 0.352114 | 0.569635 | 0.552068 | 0.611665 | 0.504045 | 0.844748 | 0.630814 | 0.72062 | 0.505673 | 0.493645 | 0.833256 | 0.366589 |
| 25 | 0.239628 | 0.321967 | 0.286922 | 0.578549 | 0.471375 | 0.428686 | 0.45798 | 0.423925 | 0.358277 | 0.729916 | 0.558307 | 0.185438 | 0.697991 | 0.224671 |
| 30 | 0.206866 | 0.228497 | 0.38681 | 0.277604 | 0.360693 | 0.425267 | 0.38311 | 0.882037 | 0.597513 | 0.299611 | 0.529744 | 0.172337 | 0.438761 | 0.213154 |
| 35 | 0.235694 | 0.283372 | 0.315505 | 0.374385 | 0.36425 | 0.237196 | 0.531484 | 0.594472 | 0.42038 | 0.362344 | 0.200869 | 0.200481 | 0.224995 | 0.201174 |
| 40 | 0.097727 | 0.180025 | 0.138909 | 0.127293 | 0.303833 | 0.223923 | 0.402172 | 0.469573 | 0.392903 | 0.312749 | 0.206009 | 0.255466 | 0.423213 | 0.135058 |
| 45 | 0.259834 | 0.26839 | 0.357492 | 0.13488 | 0.340476 | 0.447507 | 0.19782 | 0.397036 | 0.306792 | 0.413619 | 0.317369 | 0.19601 | 0.238058 | 0.16359 |
| 50 | 0.162582 | 0.380241 | 0.321203 | 0.173221 | 0.213403 | 0.305739 | 0.312413 | 0.196595 | 0.180503 | 0.283475 | 0.368326 | 0.221662 | 0.156761 | 0.1203 |
| 55 | 0.157914 | 0.185315 | 0.10333 | 0.002509 | 0.149452 | 0.072851 | 0.072912 | 0.140763 | 0.160579 | 0.125598 | 0.159018 | 0 | 0 | 0.069666 |
| 60 | 0.230859 | 0.222232 | 0.09706 | 0.24197 | 0.214048 | 0.232664 | 0.157504 | 0.13404 | 0.225714 | 0.12943 | 0.09542 | 0 | 0.054937 | 0.125239 |
| 65 | 0.172338 | 0.23053 | 0.228917 | 0.178854 | 0.195066 | 0.15513 | 0.158499 | 0.248258 | 0.149207 | 0.184238 | 0.151683 | 0.122443 | 0.259423 | 0.164274 |

Work; urban; physical or ≥15 mins

|  | 0 | 5 | 10 | 15 | 20 | 25 | 30 | 35 | 40 | 45 | 50 | 55 | 60 | 65 |
| --- | --- | --- | --- | --- | --- | --- | --- | --- | --- | --- | --- | --- | --- | --- |
| 0 | 0 | 0 | 0 | 0.006762 | 0 | 0.003019 | 0.006038 | 0.003019 | 0 | 0 | 0 | 0 | 0 | 0 |
| 5 | 0 | 0.015312 | 0 | 0 | 0.014364 | 0 | 0 | 0.003828 | 0 | 0 | 0 | 0 | 0 | 0 |
| 10 | 0 | 0 | 0 | 0.004677 | 0.015458 | 0.007716 | 0.003286 | 0.012855 | 0 | 0.003286 | 0 | 0 | 0 | 0 |
| 15 | 0.008035 | 0 | 0.005156 | 0.174609 | 0.124434 | 0.115844 | 0.028961 | 0.036379 | 0.008035 | 0 | 0 | 0 | 0 | 0.00707 |
| 20 | 0 | 0.020075 | 0.021604 | 0.157753 | 0.210274 | 0.301352 | 0.125868 | 0.121202 | 0.089612 | 0 | 0.034133 | 0.18526 | 0.018629 | 0.008963 |
| 25 | 0.003862 | 0 | 0.009157 | 0.124707 | 0.255889 | 0.425468 | 0.264819 | 0.140363 | 0.045804 | 0.182164 | 0.067124 | 0.03801 | 0.031788 | 0.00761 |
| 30 | 0.007645 | 0 | 0.00386 | 0.030861 | 0.105794 | 0.262131 | 0.144585 | 0.183136 | 0.170203 | 0.302071 | 0.229514 | 0.037624 | 0.062931 | 0.014286 |
| 35 | 0.002137 | 0.002514 | 0.008441 | 0.021669 | 0.056944 | 0.077663 | 0.102369 | 0.118178 | 0.16963 | 0.194223 | 0.146021 | 0.029472 | 0.052059 | 0.008441 |
| 40 | 0 | 0 | 0 | 0.005928 | 0.052145 | 0.031388 | 0.117833 | 0.210091 | 0.150765 | 0.206172 | 0.059585 | 0.116752 | 0.087135 | 0.031292 |
| 45 | 0 | 0 | 0.002197 | 0 | 0 | 0.102641 | 0.171948 | 0.197786 | 0.169519 | 0.111774 | 0.048992 | 0.060224 | 0.017911 | 0 |
| 50 | 0 | 0 | 0 | 0 | 0.015007 | 0.034754 | 0.120054 | 0.136643 | 0.04502 | 0.04502 | 0.18008 | 0.04502 | 0.030013 | 0.015762 |
| 55 | 0 | 0 | 0 | 0 | 0.063883 | 0.015435 | 0.015435 | 0.021631 | 0.069187 | 0.043405 | 0.03531 | 0 | 0.038727 | 0.003091 |
| 60 | 0 | 0 | 0 | 0 | 0.004978 | 0.010004 | 0.020009 | 0.029612 | 0.040018 | 0.010004 | 0.018243 | 0.030013 | 0 | 0 |
| 65 | 0 | 0 | 0 | 0.004172 | 0.004172 | 0.004172 | 0.007912 | 0.008364 | 0.025033 | 0 | 0.016689 | 0.004172 | 0 | 0 |

Work; rural; physical or ≥15 mins

|  | 0 | 5 | 10 | 15 | 20 | 25 | 30 | 35 | 40 | 45 | 50 | 55 | 60 | 65 |
| --- | --- | --- | --- | --- | --- | --- | --- | --- | --- | --- | --- | --- | --- | --- |
| 0 | 0 | 0.003154 | 0 | 0.003438 | 0.003438 | 0 | 0.003438 | 0 | 0.021282 | 0 | 0 | 0 | 0 | 0 |
| 5 | 0.00357 | 0.014282 | 0.005519 | 0.002512 | 0.00357 | 0 | 0.00357 | 0 | 0.027659 | 0 | 0 | 0.007141 | 0 | 0 |
| 10 | 0 | 0.005894 | 0.064835 | 0.025204 | 0.002947 | 0 | 0 | 0.040535 | 0.002947 | 0 | 0.002947 | 0 | 0 | 0 |
| 15 | 0.004118 | 0.002659 | 0.024975 | 0.062829 | 0.005628 | 0.044089 | 0.005937 | 0 | 0.025492 | 0 | 0 | 0.068632 | 0.016841 | 0 |
| 20 | 0.004118 | 0.003778 | 0.00292 | 0.005628 | 0 | 0.050073 | 0 | 0.017163 | 0.025492 | 0.030903 | 0 | 0.068632 | 0.016841 | 0 |
| 25 | 0 | 0 | 0 | 0.033977 | 0.038589 | 0.015435 | 0.015435 | 0.068342 | 0.078361 | 0.023815 | 0.035904 | 0.170985 | 0.012978 | 0 |
| 30 | 0.002947 | 0.002704 | 0 | 0.004248 | 0 | 0.014333 | 0.015659 | 0.061409 | 0.00783 | 0.088457 | 0 | 0.378089 | 0.061662 | 0 |
| 35 | 0 | 0 | 0.017319 | 0 | 0.007401 | 0.038239 | 0.037003 | 0.029602 | 0.04358 | 0.028126 | 0.014801 | 0.073477 | 0.076037 | 0 |
| 40 | 0.012278 | 0.014098 | 0.001407 | 0.012278 | 0.012278 | 0.048975 | 0.00527 | 0.04868 | 0 | 0.103428 | 0 | 0.07961 | 0.153336 | 0 |
| 45 | 0 | 0 | 0 | 0 | 0.014601 | 0.014601 | 0.058404 | 0.030819 | 0.101458 | 0 | 0 | 0.014601 | 0.089809 | 0 |
| 50 | 0 | 0 | 0.001232 | 0 | 0 | 0.019662 | 0 | 0.014486 | 0 | 0 | 0.061742 | 0 | 0.134352 | 0.015435 |
| 55 | 0 | 0.002288 | 0 | 0.020778 | 0.020778 | 0.067173 | 0.159961 | 0.05159 | 0.05004 | 0.009356 | 0 | 0 | 0.138744 | 0 |
| 60 | 0 | 0 | 0 | 0.004326 | 0.004326 | 0.004326 | 0.022135 | 0.045299 | 0.081779 | 0.048828 | 0.081779 | 0.117723 | 0.04089 | 0.061335 |
| 65 | 0 | 0 | 0 | 0 | 0 | 0 | 0 | 0 | 0 | 0 | 0.019462 | 0 | 0.12705 | 0 |

School; urban; physical or ≥15 mins

|  | 0 | 5 | 10 | 15 | 20 | 25 | 30 | 35 | 40 | 45 | 50 | 55 | 60 | 65 |
| --- | --- | --- | --- | --- | --- | --- | --- | --- | --- | --- | --- | --- | --- | --- |
| 0 | 0 | 0.003551 | 0 | 0 | 0 | 0 | 0 | 0 | 0 | 0 | 0 | 0 | 0.049699 | 0 |
| 5 | 0.003828 | 1.615133 | 0.309667 | 0.030319 | 0.030424 | 0.007656 | 0.034479 | 0.007656 | 0.016204 | 0.007656 | 0.003828 | 0 | 0 | 0 |
| 10 | 0 | 0.309667 | 2.275104 | 0.17854 | 0.026289 | 0.023002 | 0.026289 | 0.178322 | 0.027054 | 0.01796 | 0.006572 | 0.013909 | 0 | 0 |
| 15 | 0 | 0.033424 | 0.196824 | 1.313151 | 0.302712 | 0.032739 | 0.007735 | 0.016069 | 0.019248 | 0.010913 | 0 | 0.005757 | 0.029529 | 0 |
| 20 | 0 | 0.04252 | 0.036741 | 0.383768 | 0.396591 | 0.064387 | 0.017939 | 0.008801 | 0 | 0.002934 | 0.013865 | 0.013865 | 0 | 0 |
| 25 | 0 | 0.009086 | 0.027298 | 0.035244 | 0.054673 | 0.017305 | 0.034792 | 0 | 0 | 0 | 0 | 0 | 0.031788 | 0 |
| 30 | 0 | 0.040503 | 0.030881 | 0.008242 | 0.015078 | 0.034438 | 0.094542 | 0.015101 | 0.021831 | 0 | 0 | 0 | 0.062931 | 0 |
| 35 | 0 | 0.005027 | 0.117091 | 0.009571 | 0.004135 | 0 | 0.008441 | 0 | 0 | 0 | 0 | 0 | 0 | 0 |
| 40 | 0 | 0.013178 | 0.022001 | 0.014199 | 0 | 0 | 0.015114 | 0 | 0 | 0 | 0 | 0 | 0.021784 | 0 |
| 45 | 0 | 0.00512 | 0.01201 | 0.006619 | 0.001404 | 0 | 0 | 0 | 0 | 0 | 0 | 0 | 0 | 0 |
| 50 | 0 | 0.002352 | 0.004038 | 0 | 0.006096 | 0 | 0 | 0 | 0 | 0 | 0 | 0 | 0 | 0 |
| 55 | 0 | 0 | 0.006703 | 0.002517 | 0.004781 | 0 | 0 | 0 | 0 | 0 | 0 | 0 | 0.012909 | 0 |
| 60 | 0.020009 | 0 | 0 | 0.010004 | 0 | 0.010004 | 0.020009 | 0 | 0.010004 | 0 | 0 | 0.010004 | 0 | 0 |
| 65 | 0 | 0 | 0 | 0 | 0 | 0 | 0 | 0 | 0 | 0 | 0 | 0 | 0 | 0 |

School; rural; physical or ≥15 mins

|  | 0 | 5 | 10 | 15 | 20 | 25 | 30 | 35 | 40 | 45 | 50 | 55 | 60 | 65 |
| --- | --- | --- | --- | --- | --- | --- | --- | --- | --- | --- | --- | --- | --- | --- |
| 0 | 0.006876 | 0.10368 | 0.023067 | 0.004439 | 0 | 0 | 0 | 0.132397 | 0 | 0 | 0 | 0 | 0 | 0.013241 |
| 5 | 0.117352 | 2.339448 | 0.244799 | 0.024331 | 0.014282 | 0.010711 | 0.014282 | 0.102514 | 0.010711 | 0.00357 | 0.00357 | 0 | 0 | 0.015465 |
| 10 | 0.027883 | 0.261436 | 2.42389 | 0.258233 | 0 | 0.005894 | 0.011788 | 0.005894 | 0.008841 | 0.008841 | 0 | 0.005894 | 0 | 0 |
| 15 | 0.005318 | 0.025749 | 0.255885 | 0.965461 | 0.102141 | 0 | 0.005318 | 0 | 0 | 0 | 0 | 0 | 0.002659 | 0 |
| 20 | 0 | 0.015114 | 0 | 0.102141 | 0.06993 | 0.003496 | 0 | 0.003496 | 0.050984 | 0 | 0 | 0 | 0 | 0 |
| 25 | 0 | 0.008735 | 0.004501 | 0 | 0.002695 | 0 | 0 | 0 | 0 | 0 | 0 | 0 | 0 | 0.006111 |
| 30 | 0 | 0.010815 | 0.008359 | 0.003805 | 0 | 0 | 0 | 0 | 0.018242 | 0 | 0 | 0 | 0 | 0.005675 |
| 35 | 0.068381 | 0.046778 | 0.002518 | 0 | 0.001508 | 0 | 0 | 0 | 0.010992 | 0 | 0 | 0 | 0 | 0 |
| 40 | 0 | 0.00546 | 0.00422 | 0 | 0.024556 | 0 | 0.012278 | 0.012278 | 0.098226 | 0 | 0 | 0 | 0 | 0 |
| 45 | 0 | 0.001785 | 0.004139 | 0 | 0 | 0 | 0 | 0 | 0 | 0 | 0 | 0 | 0 | 0 |
| 50 | 0 | 0.001595 | 0 | 0 | 0 | 0 | 0 | 0 | 0 | 0 | 0 | 0 | 0 | 0 |
| 55 | 0 | 0 | 0.001768 | 0 | 0 | 0 | 0 | 0 | 0 | 0 | 0 | 0 | 0 | 0 |
| 60 | 0 | 0 | 0 | 0.000683 | 0 | 0 | 0 | 0 | 0 | 0 | 0 | 0 | 0 | 0.002037 |
| 65 | 0.008439 | 0.008709 | 0 | 0 | 0 | 0.00422 | 0.00422 | 0 | 0 | 0 | 0 | 0 | 0.00422 | 0 |

Other (including transport and leisure); urban; physical or ≥15 mins

|  | 0 | 5 | 10 | 15 | 20 | 25 | 30 | 35 | 40 | 45 | 50 | 55 | 60 | 65 |
| --- | --- | --- | --- | --- | --- | --- | --- | --- | --- | --- | --- | --- | --- | --- |
| 0 | 0.140275 | 0.093569 | 0.108686 | 0.042142 | 0.061531 | 0.044558 | 0.033909 | 0.038704 | 0.039186 | 0.018816 | 0.016742 | 0.042492 | 0.012076 | 0.015709 |
| 5 | 0.100861 | 0.377138 | 0.214869 | 0.049418 | 0.005927 | 0.059073 | 0.011484 | 0.045743 | 0.019277 | 0.008547 | 0 | 0.068185 | 0.040912 | 0.010241 |
| 10 | 0.117156 | 0.214869 | 0.367587 | 0.075475 | 0.073519 | 0.026289 | 0.037786 | 0.049478 | 0.038451 | 0.009858 | 0.01796 | 0.103181 | 0.0493 | 0.007337 |
| 15 | 0.050078 | 0.054478 | 0.083204 | 0.389642 | 0.324442 | 0.195741 | 0.116244 | 0.212798 | 0.171692 | 0.109937 | 0.106186 | 0.060025 | 0.037564 | 0.008035 |
| 20 | 0.092697 | 0.008284 | 0.10275 | 0.411315 | 0.323692 | 0.294279 | 0.138804 | 0.272275 | 0.167371 | 0.150136 | 0.344847 | 0.002934 | 0.267655 | 0.041718 |
| 25 | 0.057 | 0.070105 | 0.031198 | 0.210716 | 0.249883 | 0.601939 | 0.407595 | 0.354057 | 0.192505 | 0.22266 | 0.117494 | 0.133484 | 0.185035 | 0.030442 |
| 30 | 0.042937 | 0.013491 | 0.044387 | 0.123867 | 0.116667 | 0.403458 | 0.248002 | 0.43805 | 0.214484 | 0.104561 | 0.184366 | 0.431531 | 0.197645 | 0.051172 |
| 35 | 0.027395 | 0.030036 | 0.032489 | 0.126749 | 0.127922 | 0.1959 | 0.244859 | 0.219354 | 0.250803 | 0.184834 | 0.162885 | 0.231457 | 0.117935 | 0.034687 |
| 40 | 0.034351 | 0.015677 | 0.031271 | 0.126658 | 0.097393 | 0.131919 | 0.148489 | 0.310627 | 0.212892 | 0.173501 | 0.139086 | 0.038353 | 0.209325 | 0.02821 |
| 45 | 0.013562 | 0.005715 | 0.006592 | 0.066683 | 0.071833 | 0.125458 | 0.059519 | 0.188225 | 0.142657 | 0.335322 | 0.081019 | 0.037258 | 0.214419 | 0.017152 |
| 50 | 0.011089 | 0 | 0.011036 | 0.059186 | 0.151614 | 0.060835 | 0.096437 | 0.152424 | 0.105087 | 0.07445 | 0 | 0.034687 | 0.296347 | 0.048889 |
| 55 | 0.022074 | 0.03286 | 0.049726 | 0.026241 | 0.001012 | 0.054207 | 0.177039 | 0.169877 | 0.022728 | 0.026853 | 0.027205 | 0.029171 | 0.116028 | 0.026947 |
| 60 | 0.004862 | 0.015281 | 0.018413 | 0.012727 | 0.071529 | 0.058235 | 0.062841 | 0.067082 | 0.096135 | 0.119766 | 0.180133 | 0.089921 | 0.204606 | 0.148659 |
| 65 | 0.011017 | 0.006663 | 0.004774 | 0.004742 | 0.01942 | 0.016689 | 0.028341 | 0.034368 | 0.022568 | 0.016689 | 0.051765 | 0.036379 | 0.258952 | 0.141855 |

Other (including transport and leisure); rural; physical or ≥15 mins

|  | 0 | 5 | 10 | 15 | 20 | 25 | 30 | 35 | 40 | 45 | 50 | 55 | 60 | 65 |
| --- | --- | --- | --- | --- | --- | --- | --- | --- | --- | --- | --- | --- | --- | --- |
| 0 | 0.340145 | 0.54131 | 0.405275 | 0.236227 | 0.190277 | 0.210606 | 0.157428 | 0.161971 | 0.172448 | 0.122897 | 0.133036 | 0.195962 | 0.095008 | 0.087378 |
| 5 | 0.612691 | 1.251504 | 0.516786 | 0.323366 | 0.33305 | 0.270018 | 0.249311 | 0.271914 | 0.069427 | 0.047054 | 0.424764 | 0.262987 | 0.148875 | 0.114128 |
| 10 | 0.489893 | 0.551908 | 0.977913 | 0.485485 | 0.357706 | 0.241395 | 0.319445 | 0.377285 | 0.302261 | 0.357225 | 0.22876 | 0.274315 | 0.133425 | 0.141949 |
| 15 | 0.282954 | 0.342203 | 0.481072 | 0.806042 | 0.781601 | 0.748625 | 0.37784 | 0.221517 | 0.397729 | 0.657495 | 0.739327 | 0.150466 | 0.162626 | 0.12239 |
| 20 | 0.227914 | 0.352451 | 0.354454 | 0.781601 | 1.268067 | 1.055407 | 0.824074 | 0.753648 | 0.674508 | 1.075649 | 0.748002 | 0.614104 | 0.206915 | 0.217392 |
| 25 | 0.194406 | 0.220209 | 0.184338 | 0.576922 | 0.813342 | 0.826415 | 0.732862 | 0.709973 | 0.56665 | 0.760001 | 0.636835 | 0.539038 | 0.469535 | 0.170654 |
| 30 | 0.134938 | 0.188799 | 0.226515 | 0.270381 | 0.589704 | 0.680514 | 0.750562 | 0.609354 | 0.458799 | 1.028015 | 0.824226 | 0.645361 | 0.136166 | 0.163238 |
| 35 | 0.083656 | 0.124077 | 0.161203 | 0.095517 | 0.324968 | 0.397247 | 0.367175 | 0.45437 | 0.272929 | 0.682272 | 0.492843 | 0.181025 | 0.128377 | 0.102766 |
| 40 | 0.099489 | 0.035388 | 0.144261 | 0.191567 | 0.324878 | 0.354156 | 0.308807 | 0.304867 | 0.27012 | 0.326413 | 0.433091 | 0.263509 | 0.199346 | 0.115892 |
| 45 | 0.069552 | 0.023527 | 0.167246 | 0.310651 | 0.508219 | 0.465953 | 0.678753 | 0.747595 | 0.320195 | 0.123607 | 0.438064 | 0.22306 | 0.30954 | 0.161984 |
| 50 | 0.067249 | 0.1897 | 0.095663 | 0.312009 | 0.31567 | 0.348742 | 0.486082 | 0.482356 | 0.37947 | 0.39128 | 0.778959 | 0.435345 | 0.340541 | 0.453832 |
| 55 | 0.071063 | 0.084258 | 0.082294 | 0.045554 | 0.185921 | 0.211765 | 0.273037 | 0.127102 | 0.165634 | 0.142932 | 0.312312 | 0.444592 | 0.225733 | 0.148723 |
| 60 | 0.029233 | 0.040471 | 0.033963 | 0.041775 | 0.053153 | 0.156512 | 0.04888 | 0.07648 | 0.106318 | 0.168294 | 0.207286 | 0.191531 | 0.174709 | 0.245346 |
| 65 | 0.055691 | 0.064266 | 0.074846 | 0.065125 | 0.115677 | 0.117832 | 0.121382 | 0.126818 | 0.128033 | 0.182429 | 0.572224 | 0.261393 | 0.508217 | 0.313635 |
